# *Pseudomonas aeruginosa* Pili and Flagella Mediate Distinct Binding and Signaling Events at the Apical and Basolateral Surface of Airway Epithelium

**DOI:** 10.1371/journal.ppat.1002616

**Published:** 2012-04-05

**Authors:** Iwona Bucior, Julia F. Pielage, Joanne N. Engel

**Affiliations:** 1 Department of Medicine, University of California San Francisco, San Francisco, California, United States of America; 2 Microbial Pathogenesis and Host Defense Program, University of California San Francisco, San Francisco, California, United States of America; 3 Department of Microbiology and Immunology, University of California San Francisco, San Francisco, California, United States of America; Medical College of Wisconsin, United States of America

## Abstract

*Pseudomonas aeruginosa*, an important opportunistic pathogen of man, exploits numerous factors for initial attachment to the host, an event required to establish bacterial infection. In this paper, we rigorously explore the role of two major bacterial adhesins, type IV pili (Tfp) and flagella, in bacterial adherence to distinct host receptors at the apical (AP) and basolateral (BL) surfaces of polarized lung epithelial cells and induction of subsequent host signaling and pathogenic events. Using an isogenic mutant of *P. aeruginosa* that lacks flagella or utilizing beads coated with purified Tfp, we establish that Tfp are necessary and sufficient for maximal binding to host N-glycans at the AP surface of polarized epithelium. In contrast, experiments utilizing a *P. aeruginosa* isogenic mutant that lacks Tfp or using beads coated with purified flagella demonstrate that flagella are necessary and sufficient for maximal binding to heparan sulfate (HS) chains of heparan sulfate proteoglycans (HSPGs) at the BL surface of polarized epithelium. Using two different cell-free systems, we demonstrate that Tfp-coated beads show highest binding affinity to complex N-glycan chains coated onto plastic plates and preferentially aggregate with beads coated with N-glycans, but not with single sugars or HS. In contrast, flagella-coated beads bind to or aggregate preferentially with HS or HSPGs, but demonstrate little binding to N-glycans. We further show that Tfp-mediated binding to host N-glycans results in activation of phosphatidylinositol 3-kinase (PI3K)/Akt pathway and bacterial entry at the AP surface. At the BL surface, flagella-mediated binding to HS activates the epidermal growth factor receptor (EGFR), adaptor protein Shc, and PI3K/Akt, and induces bacterial entry. Remarkably, flagella-coated beads alone can activate EGFR and Shc. Together, this work provides new insights into the intricate interactions between *P. aeruginosa* and lung epithelium that may be potentially useful in the development of novel treatments for *P. aeruginosa* infections.

## Author Summary

*Pseudomonas aeruginosa* is one of the most virulent nosocomial opportunistic pathogens that is associated with a broad spectrum of life-threatening infections. Antibiotic resistance is widespread and attributable mortality remains near 50%. Complex binding to epithelial cells is a key first step for this potent pathogen to unleash its armamentarium of virulence factors. Polarized epithelium has distinct apical (AP) and basolateral (BL) surface, composed of different glycosylated molecules, and *P. aeruginosa* can potentially employ different adhesins to bind to these receptors. Using isogenic mutants as well as *in vitro* cell-free assays, we demonstrate that bacterial type IV pili are necessary and sufficient to mediate AP interactions with N-glycans whereas bacterial flagella interact with heparan sulfate chains of proteoglycans on the BL surface. These interactions induce specific host signaling pathways that lead to subsequent pathogenic events, such as bacterial entry into host epithelium. Moreover, we show that flagella alone are sufficient to activate the epidermal growth factor receptor and the adaptor protein on the BL surface. These studies reveal new information about key players in the versatile interactions of *P. aeruginosa* with the host and provide appealing targets for blocking early binding steps essential for establishment of *P. aeruginosa* infections.

## Introduction

*Pseudomonas aeruginosa* is an opportunistic human pathogen associated with a broad spectrum of life-threatening infections in the setting of epithelial injury and immunocompromise (reviewed in [1]). This gram-negative pathogen ranks among the leading causes of hospital-acquired pneumonia, urinary tract infections, bloodstream infections, and surgical site infections. In addition to their frequent occurrence, nosocomial *P. aeruginosa* infections are often severe, with an excess attributable mortality rate of almost 50% for mechanically ventilated patients with *P. aeruginosa* pneumonia [2]. The bacterium is also the leading cause of respiratory morbidity and mortality in patients with cystic fibrosis (CF) [3], [4], as well as a frequent cause of exacerbations in individuals with advanced chronic obstructive pulmonary disease [5]. *P. aeruginosa* infections are also reported as a complication of HIV infections and are becoming more frequent as patients with AIDS survive longer [6]–[8]. Notably, therapeutic options are becoming increasingly limited with the continued emergence and spread of multi-drug resistant strains. Thus, increasing our understanding of the pathogenesis of *P. aeruginosa* infections is critical for the development of new therapeutics that target this medically important pathogen.

The pathogenesis of *P. aeruginosa* infections is multifactorial and complex. Bacterial attachment is an initial and critical step that involves complex interactions between bacterial adhesins and host receptors either on the apical (AP) or basolateral (BL) surface of polarized epithelium. Using cultured epithelial cells grown as polarized monolayers, which recapitulate simple epithelial tissue, or as three-dimensional cysts, which mimic the organization of simple epithelial organs, we have recently demonstrated that N-glycans are necessary and sufficient for bacterial binding and consequent entry and cytotoxicity at the AP surface of polarized epithelium [9]. In contrast, heparan sulfate (HS) chains of heparan sulfate proteoglycans (HSPGs) are necessary and sufficient to mediate these events at the BL surface of polarized cells. We showed that in incompletely polarized cells, a model for tissue injury, HSPGs are upregulated at the AP surface, which leads to enhanced binding and subsequent tissue damage by *P. aeruginosa*. These results provide an explanation, at least to some extent, for the increased susceptibility of injured tissue to *P. aeruginosa* infections. Although our previous work characterized distinct AP and BL host receptors, the downstream host signaling pathways associated with these critical events and bacterial binding partners remained to be elucidated.

Two major adhesins have been identified in *P. aeruginosa*, flagella and type IV pili (Tfp) [10]–[12]. The single polar flagellum is a polymer composed of flagellin, the product of the *fliC* gene, although a large number of gene products are required for flagellar assembly and function [13]. Flagella are required for adhesion to cells, swimming motility, and biofilm formation. In addition, monomeric flagellin is recognized by the innate immune system, either by binding to Toll-like receptor 5 (TLR5) at the cell surface or by recognition of individual subunits by intracellular cytosolic sensors [14]–[18] Mechanistic or structural details of the interaction of flagella with the host epithelium are still lacking. The flagellar cap protein of *P. aeruginosa* strain O1 (PAO1), but not other strains, binds to Lewis^X^ oligosaccharides in mucins [19], but whether this is relevant to binding to host epithelial cells is unknown.

Tfp are polarly localized appendages composed of pilin polymers that undergo reversible assembly and disassembly, allowing the bacteria to move over a solid surface in a process termed twitching motility (reviewed in [20]). Tfp also function as phage receptors, contribute to early steps in biofilm formation, and serve as adhesins to mammalian cells [21]. Several studies have identified different glycosphingolipids as host receptors for Tfp-mediated binding at the AP surface of polarized cells [22], [23], although their roles in mediating bacterial binding remain controversial [24], [25].

Following adhesion to host epithelium, *P. aeruginosa* can induce host cell death or enter non-phagocytic cells (reviewed in [10], [26]). Internalization may permit the bacteria to penetrate the epithelial cell layer, reach the bloodstream, and disseminate to distant organs and/or it may represent a host defense mechanism that contributes to bacterial clearance [10], [27]. The molecular events underlying *P. aeruginosa* invasion into non-phagocytic cells are incompletely understood. *P. aeruginosa* entry is an actin-dependent process that involves Rho family GTPases [28], activation of tyrosine kinases, such as Src [29], [30] or Abl [31] kinases and subsequent tyrosine phosphorylation of several host proteins, including caveolin [32]. We have previously shown that phosphatidylinositol 3-kinase (PI3K) and its effector protein Akt (also known as the serine threonine protein kinase B) are necessary and sufficient for and are activated upon bacterial internalization into Madin Darby Canine Kidney (MDCK) cells [33]. However, specific AP or BL upstream receptors associated with this event have not yet been identified, and the PI3K/Akt pathway can be activated by many stimuli, including growth factor receptors, such as the epidermal growth factor receptor (EGFR) [34]. Furthermore, the role of bacterial ligands, e.g. Tfp or flagella, in these newly described signaling events has also not been investigated.

In this work, we characterize important bacterial and host factors that play an essential role in the complex binding and signaling networks utilized by *P. aeruginosa*. We rigorously assess the role of Tfp and flagella in mediating bacterial binding to specific host N-glycans and HSPGs at the AP and BL surfaces, and test whether such interactions dictate activation of specific signaling pathways. We demonstrate that Tfp are necessary and sufficient to mediate maximal bacterial binding to N-glycans at the AP surface, while flagella are necessary and sufficient to mediate maximal bacterial binding to HS chains of HSPGs at the BL surface of polarized airway epithelium. We find that *P. aeruginosa* internalization at the AP surface is dependent on Tfp binding to N-glycans and, to some extent, on activation of PI3K and Akt. *P. aeruginosa* internalization at the BL surface is dependent on flagella binding to HS followed by activation of EGFR and PI3K/Akt pathway. Remarkably, flagella-coated beads alone are sufficient to trigger EGFR phosphorylation and activation of downstream adaptor protein. Our work identifies key factors and interactions required for establishing *P. aeruginosa* attachment and internalization, affording new avenues for development of treatments for acute and chronic *P. aeruginosa* infections.

## Results

### Tfp-deficient *P. aeruginosa* mutant binds preferentially to HS at the BL surface while flagella-deficient mutant binds preferentially to N-glycans at the AP surface of polarized epithelium

Our previous studies established that *P. aeruginosa* binds preferentially to N-glycan chains at the AP surface of polarized epithelium, with preferential affinity for more complex chains after up-regulations of N-glycosylation [9]. At the BL surface, the bacterium binds preferably to HS chains of HSPGs. To determine whether flagella or Tfp, the major *P. aeruginosa* adhesins, differentially mediate binding to these distinct AP and BL host receptors, we utilized isogenic mutants of PAO1 in which the gene encoding PilA, the major subunit of the Tfp (PAO1Δ*pilA*) or the gene encoding FliC, the major subunit of flagella (PAO1Δ*fliC*), is deleted. Standard adhesion assays, in which bacteria were added for 2 h to the AP or BL surface of polarized Calu-3 cells grown as polarized monolayers on Transwell filters, were performed [9]. While other *P. aeruginosa* adhesins have been identified, such as the cup fimbrial adhesins [35] and lectins PA-IL (LecA) and PA-IIL (LecB) [36], Tfp and flagella were the predominant adhesins under the conditions of our experiments, as the PAO1Δ*fliC*Δ*pilA* double mutant exhibited undetectable binding (data not shown and [21]).

**Figure 1:**
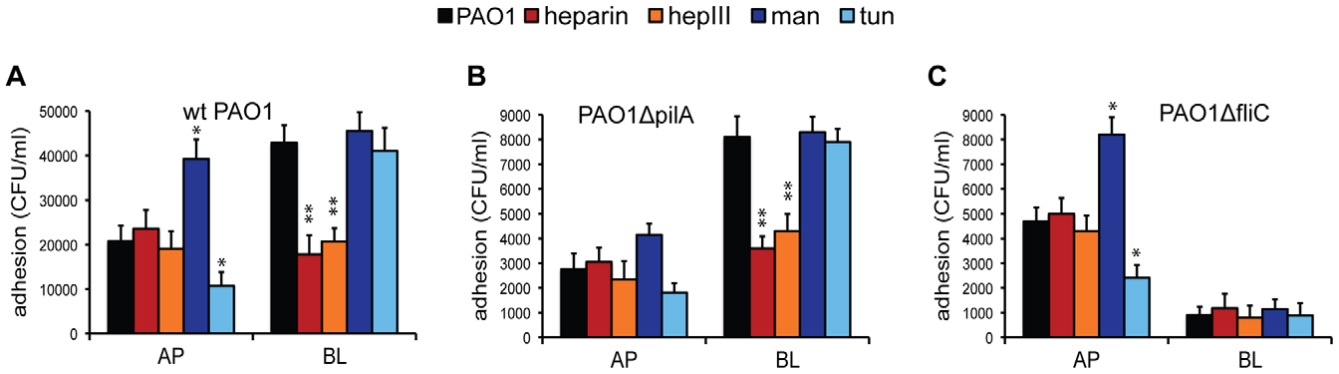
PAO1Δ*pilA* binds preferentially to HS at the BL surface and PAO1Δ*fliC* binds preferentially to N-glycans at the AP surface. Calu-3 cells were grown as well polarized monolayers on Transwells for 9 days and treated with heparin, heparinase III (hepIII), mannose (Man), or tunicamycin (tun). Host cell treatments are color-coded: treatments that affect HSPGs are indicated with shades of red and treatments that affect N-glycans are indicated with shades of blue. (**A**) PAO1, (**B**) PAO1Δ*pilA*, or (**C**) PAO1Δ*fliC* were added to the AP or BL chamber for 2 h and standard adhesion assays were performed. Shown is the mean +/− SD for 4 independent experiments. ^*^P<0.05 compared to cells infected with PAO1 at the AP surface (black bar). ^**^P<0.05 compared to cells infected with PAO1 at the BL surface (black bar).

Consistent with our previously published studies utilizing PAK [9], PAO1 bound approximately 2-fold more efficiently to the BL surface than to the AP surface of polarized airway epithelium (Figure 1A). Both the Tfp mutant (PAO1Δ*pilA*) and the flagella mutant (PAO1Δ*fliC*) bound less efficiently to the AP or BL surfaces of the epithelium when compared to PAO1 (Figures 1A–C), suggesting that the absence of either of these two adhesins impacts bacterial binding and that Tfp and flagella may function synergistically. Importantly, PAO1Δ*fliC* still bound almost 2-fold better than PAO1Δ*pilA* to the AP surface (P<0.05), but it bound ∼9-fold less efficiently than PAO1Δ*pilA* to the BL surface (P<0.05) (compare Figures 1B and C). Furthermore, while PAO1Δ*pilA* bound ∼3-fold more efficiently to the BL surface than to the AP surface of polarized epithelium (Figure 1B), PAO1Δ*fliC* adhered over 5-fold better to the AP surface than to the BL surface (Figure 1C). These results suggest that Tfp are the predominant adhesin at the AP surface whereas flagella function as the major adhesin at the BL surface.

We then investigated the role of host N-glycans in Tfp- or flagella-mediated binding. N-glycans are found on both AP and BL surfaces of polarized epithelium whereas HS chains are expressed predominantly on the BL surface of polarized epithelium [9]. When the expression of more complex N-glycans was triggered by long-term culture of Calu-3 cells in the presence of mannose (Man) [37], we observed a 2-fold increase in the binding of PAO1 or PAO1Δ*fliC* to the AP surface of polarized airway epithelium. However, no effect on the BL binding of PAO1 or PAO1Δ*fliC* was observed (Figure 1C). In control experiments, long-term culture of Calu-3 cells in the presence of galactose (Gal), which does not enhance N-glycosylation, had no effect on bacterial adhesion (data not shown). Inhibition of N-glycosylation by pre-treatment of Calu-3 cells with tunicamycin, which decreased N-glycosylation by 50% under the conditions of our experiments (more extensive deglycosylation disrupted the monolayer integrity), decreased the AP adhesion of PAO1 (Figure 1A) and PAO1Δ*fliC* (Figure 1C) by 2.5-fold, but had no effect on BL binding. These treatments did not have statistically significant effects on binding of PAO1Δ*pilA* to the AP surface of polarized cells (Figure 1B). Together, these results suggest that Tfp bind primarily to N-glycans at the AP surface of polarized epithelial cells.

We used two approaches to determine the role of host HS chains of HSPGs in Tfp- and flagella-mediated bacterial adherence. First, addition of excess heparin competitively inhibited the binding of PAO1 and PAO1Δ*pilA* at the BL surface of Calu-3 cells (Figures 1A and B), but had no effect on binding of PAO1Δ*fliC* to the BL surface (Figure 1C). Since HSPGs are predominantly expressed on the BL surface, it was not surprising to observe that exogenous addition of heparin had little effect on binding of PAO1 or of either adhesin mutant to the AP surface of polarized cells. To rule out non-specific charge effects, we demonstrated that addition of another highly negatively charged glycosaminoglycan chain, chondroitin sulfate (CS), had no effect on binding of PAO1, PAO1Δ*pilA*, or PAO1Δ*fliC* to either surface (data not shown). Second, pre-treatment of cells with heparinase III, an enzyme that cleaves HS chains, had a similar effect on bacterial adhesion as did addition of excess heparin. It reduced adhesion of PAO1 and PAO1Δ*pilA* to the BL surface of polarized Calu-3 cells but had no effect on adhesion of PAO1Δ*fliC*. Heparinase III treatment had no significant effect on the binding of any of the strains to the AP surface of polarized Calu-3 cells. Enzymatic removal of CS by chondroitinase ABC did not alter bacterial attachment at either surface (data not shown), confirming the specific role of HS in flagella-mediated binding of *P. aeruginosa*. Together, these results demonstrate *P. aeruginosa* binding at the BL surface is predominantly mediated by flagella interactions with HS chains of HSPGs.

### Flagella-coated beads bind directly to HS while Tfp-coated beads bind directly to N-glycans *in vitro*

Two different *in vitro* binding affinity assays were utilized to rigorously determine whether Tfp or flagella were sufficient to mediate binding to N-glycans or HS, respectively. We have previously developed a fluorometric assay that quantifies bacterial attachment to plastic wells coated with various glycans [9]. Our studies revealed that GFP-expressing PAO1 binds in a dose-dependent manner to plastic wells coated with HS or to a complex hybrid N-glycan chain ((Gal-GlcN)_4_Man_3_(GlcN)_2_), with the strongest binding observed to HS. Here, we modified this assay; in place of bacteria, we used 2-µm green fluorescent beads coated with purified flagella or Tfp, isolated by shearing from PAO1Δ*pilA* or PAO1Δ*fliC*, respectively (Figure S1). Coommasie Blue staining of the purified adhesin preparations electrophoresed on SDS-PAGE did not reveal any contaminating products (Figure S1). We note that the sheared adhesins may be composed of short polymers (i.e. flagella or Tfp) or the individual subunits (flagellin or pilin). For ease of clarity, we will refer to them as flagella or Tfp.

**Figure 2:**
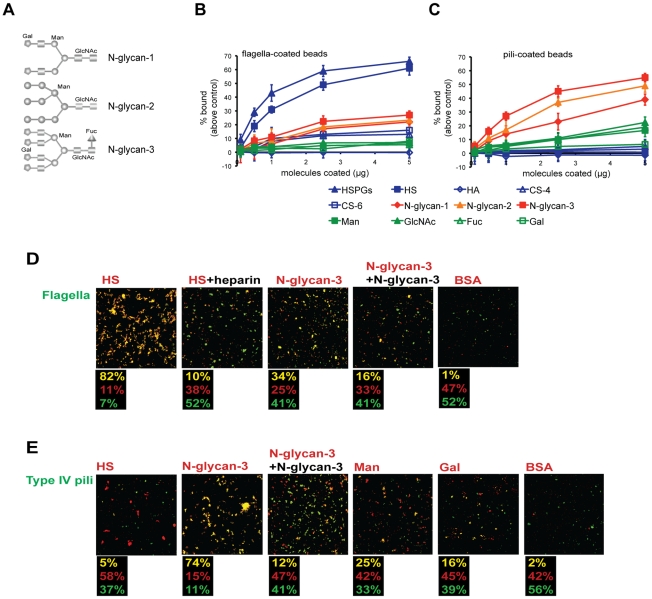
Flagella-coated beads bind directly to HS and Tfp-coated beads bind directly to N-glycans *in vitro*. (**A**) The structure of different N-glycan chains used in the study (adapted from the manufacturer). (**B**) Flagella- or (**C**) Tfp-coated beads were added to 96-well plastic plates coated with increasing concentrations of various molecules for 1 h. The fluorescence of the bound fraction was quantified in a plate reader and the percent of binding above control (binding of coated beads to non-coated wells) is indicated. Shown is the mean +/− SD for 6 independent experiments. (**D**) Flagella- or (**E**) Tfp-coated green beads were mixed with red beads coated with the indicated molecules, mixed gently for 2 h, and examined by IF. Exogenous heparin was added to competitively block flagella-HS aggregation, and exogenous N-glycan-3 to block flagella-N-glycan-3 and Tfp-N-glycan-3 aggregation. Yellow clumps indicate aggregation of green and red beads. The fraction of green aggregates, red aggregates, and mixed (yellow) aggregates from 3 separate experiments is shown beneath each panel. HSPGs: heparan sulfate proteoglycans, HS: heparan sulfate; HA: hyaluronic acid; CS-4: 4-0-sulfated chondroitin sulfate; CS-6: 6-0-sulfated chondroitin sulfate; N-glycan-1: simple N-glycan chain; N-glycan-2: hybrid N-glycan chain; N-glycan-3: complex N-glycan chain; Man: mannose; GlcNAc: N-acetylglucosamine; Fuc: fucose; Gal: galactose; BSA: bovine serum albumin.

As shown in Figure 2B, flagella-coated beads bound strongly in a dose-dependent manner to HSPG or HS chains alone and at low levels to different N-glycans (structures shown in Figure 2A), but not measurably to single sugars (Man, N-acetylated glucosamine (GlcNAc), fucose Fuc, or galactose (Gal)), CS, or to non-sulfated hyaluronic acid (HA). These results suggest that i) flagella are capable of strong binding to HS chains, that ii) sulfate groups of HS provide binding sites and/or the anionic charge of sulfate is necessary for the interaction, and that iii) flagella may also bind to sugar sequences along the N-glycan chain in a polyvalent manner.

We next examined the binding specificity of beads coated with sheared Tfp. In contrast to the results obtained with flagella-coated beads, Tfp-coated beads bound most avidly to N-glycan chains (Figure 2C), with the strongest binding to N-glycan-3, the most complex N-glycan chain (Figure 2A). There was minimal binding to the single sugars (Man, GlcNAc, Fuc, or Gal) indicating that single sugars are not sufficient to mediate binding to Tfp. Likewise, there was almost no binding to HSPGs, HS, or other glycosaminoglycans, suggesting that Tfp almost exclusively recognize N-glycan chains. Identical results were obtained with beads coated with flagella or Tfp isolated from strain PAK (Figure S2) and with pili isolated from strain PA103 (Figure S3), suggesting that the observed binding specificities are a general property of the adhesins and are not strain-specific.

As a further test for the specificity of the binding of Tfp-coated beads, we tested whether the C-terminus of Tfp, which contains the binding determinants, was required. For these experiments, we isolated sheared surface Tfp from a PA103 mutant, which harbors a transposon insertion 13 amino acids from the C-terminus (PA103 Mutant 9 [38]) (Figure S3). The mutant pilin is predicted to be truncated between the two C-terminal cysteines required for pilin folding [39]. Beads coated with Tfp isolated from PA103 pili (Figure S3) demonstrated a similar binding specificity as beads coated with Tfp from PAO1 (Figure 2) or PAK (Figure S2). In contrast, the Mutant 9 Tfp-coated beads bound poorly to N-glycans (Figure S3). These results suggest that three-dimensional structure of pili is not compromised under the conditions of our experiments and that the C-terminal binding determinants are required for *in vitro* binding of Tfp to N-glycans.

We extended our studies using a complementary *in vitro* assay that measures bead aggregation under defined shear forces to detect molecular interactions [40]. As shown in Figure 2D, we first tested the interaction of flagella-coated green fluorescent beads with red fluorescent beads coated with various glycans. In control experiments in which equal numbers of flagella-coated green beads were mixed with BSA-coated red beads, 99% of the aggregates comprised a single color (52% green and 47% red) and only 1% were yellow. These results indicate that there is minimal non-specific bead aggregation under the conditions of our experiments. In contrast, when flagella-coated green beads were mixed with HSPG-coated red beads, 80% of the aggregates were yellow. This interaction was inhibited by exogenous addition of heparin, with the number of yellow aggregates decreasing to ∼10%. Aggregation of flagella-coated green beads with N-glycan-3-coated red beads resulted in 34% yellow aggregates, which was decreased to 16% when excess N-glycan-3 was added to competitively inhibit binding. Together, both of these *in vitro* assays confirm our cell-culture based experiments and demonstrate conclusively that flagella can bind directly to HS and, to much lesser extent, to complex N-glycan chains.

We next examined the binding of Tfp-coated green beads to glycan-coated red beads (Figure 2E). Whereas Tfp-coated green beads mixed with BSA-coated red beads resulted in only 2% mixed yellow aggregates, incubation with N-glycan-3-coated red beads resulted in 74% yellow aggregates, which was decreased to 12% upon addition of excess N-glycan-3 (Figure 2E). We also tested the ability of Tfp-coated green beads to interact with red beads coated with individual sugars. Mixing Tfp-coated green beads with Man-coated red beads resulted in only 25% yellow aggregates, and in 16% yellow aggregates when Tfp-coated green beads were mixed with Gal-coated red beads. Importantly, Tfp-coated green beads did not aggregate with HS-coated red beads. These findings confirm that Tfp preferentially interact directly with complex N-glycans in a polyvalent manner and individual sugars do not provide enough strength and specificity for the interaction. Taken together, *in vitro* binding assays conclusively show that Tfp are necessary and sufficient to interact with N-glycans, which corroborates our cell-culture based experiments.

### Flagella or Tfp are sufficient to mediate binding to HS- or N-glycan-rich areas, respectively, in polarized epithelium

Our results thus far suggest that Tfp are necessary *in vivo* and sufficient *in vitro* to interact with N-glycans whereas flagella are necessary *in vivo* and sufficient *in vitro* to interact with HS. As it is possible that additional host molecules mediate bacterial binding to cultured cells, we tested by IF microscopy whether Tfp-coated or flagella-coated beads would be sufficient to mediate binding to the AP surface of airway epithelial cells and compared these results with the binding of PAO1*ΔpilA* and PAO1*ΔfliC*. For these experiments we utilized Calu-3 cells grown as confluent monolayers on Transwell filters for a shorter time period (3 days rather than the usual 9 days). Under these conditions, functional tight junctions are formed, but the polarized distribution of HSPGs is not complete, i.e. some HSPGs are still present at the AP surface [9]. These 3-day grown monolayers, which we term incompletely polarized, were briefly treated with heparinase III or tunicamycin to further decrease the surface presentation of HSPGs or N-glycans, respectively. The resulting patchy distribution of HSPGs or N-glycans at the AP surface allowed us to correlate and quantify by IF the binding of PAO1 flagella or Tfp mutants, or adhesin-coated beads, to HS-rich or -poor areas (visualized with an antibody to HS) or N-glycan-rich or -poor areas (visualized with fluorescent lectin that binds to Man residues in N-glycan chains).

**Figure 3:**
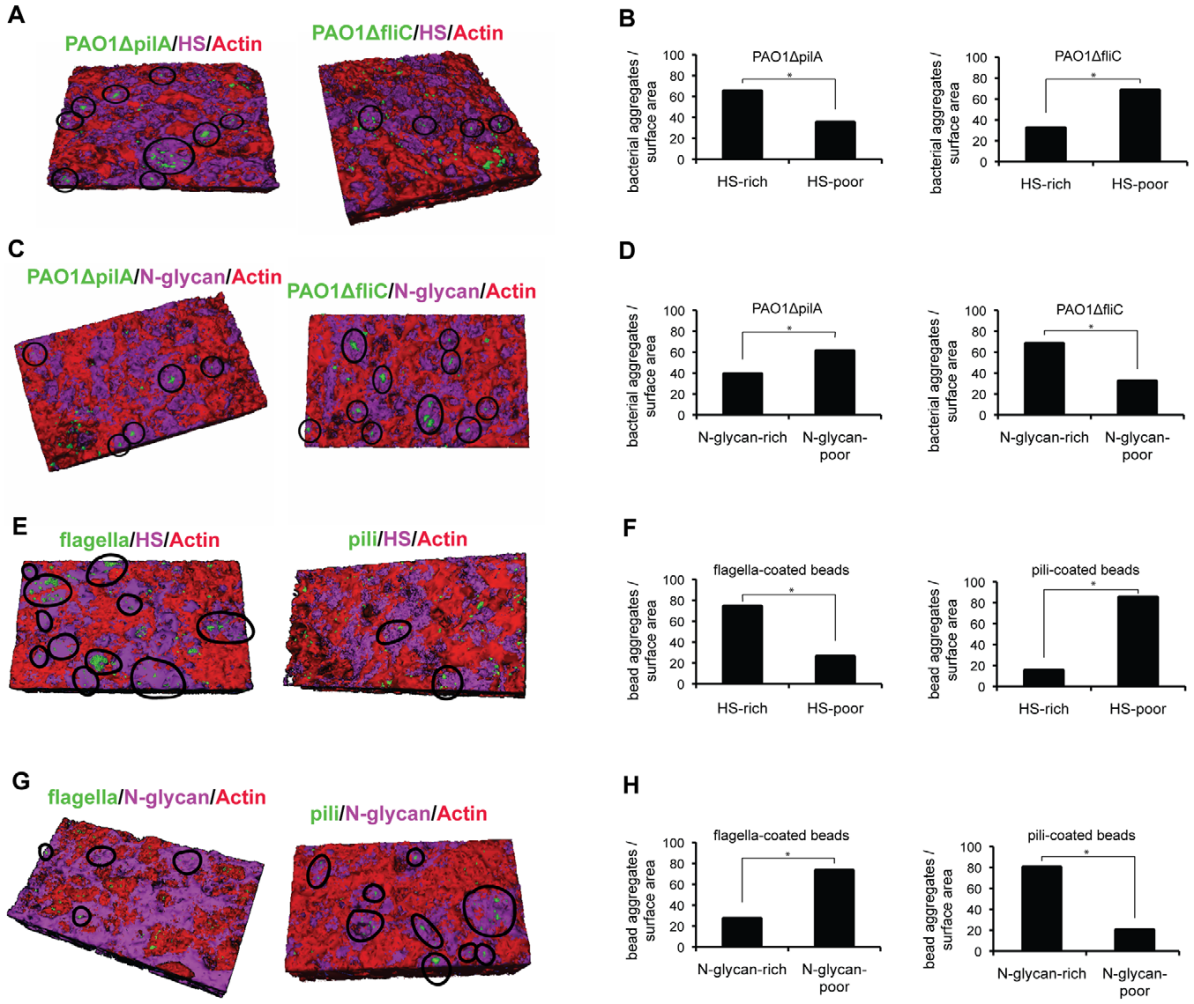
Flagella mediate co-localization with HS-rich regions while Tfp mediate co-localization with N-glycan-rich areas in polarized epithelium. Calu-3 cells were grown as incompletely polarized monolayers on Transwell filters for 3 days and briefly treated with heparinase III or with tunicamycin. Binding of bacteria or adhesin-coated beads to the AP surface was examined by confocal microscopy with 3D reconstructions of Z stack images and quantification of bacterial or coated-bead localization to HS-rich, HS-poor, N-glycan-rich, or N-glycan-poor regions of the AP surface was determined. HS was visualized with an anti-HS antibody (purple), N-glycans with concanavalin A (purple), actin with phalloidin (red). (**A**) Co-localization and (**B**) quantification of PAO1*ΔpilA*-GFP or PAO1Δ*fliC*-GFP binding to HS-rich (purple) or HS-poor (red) areas. Black circles show co-localization of *P. aeruginosa* mutants with HS-rich areas. (**C**) Co-localization and (**D**) quantification of PAO1Δ*pilA*-GFP or PAO1Δ*fliC*-GFP binding to N-glycan-rich (purple) or N-glycan-poor (red) areas. Black circles show co-localization of *P. aeruginosa* mutants with N-glycan-rich areas. (**E**) Co-localization and (**F**) quantification of flagella- or Tfp-coated green beads binding to HS-rich or HS-poor areas. Black circles show co-localization of flagella- or Tfp-coated green beads with HS-rich areas. (**G**) Co-localization and (**H**) quantification of flagella- or Tfp-coated green beads binding to N-glycan-rich (purple) or N-glycan-poor (red) areas. Black circles show co-localization of flagella- or Tfp-coated green beads with N-glycan-rich areas. Shown is the mean +/− SD for 3 independent experiments. ^*^P<0.01.

We first examined the binding of GFP-expressing PAO1Δ*pilA* and PAO1Δ*fliC* to membrane regions rich in HS or N-glycan chains. As shown in Figures 3A and B, significantly more PAO1Δ*pilA*-GFP co-localized to HS-rich patches (65%) than to HS-poor patches (35%) on the AP surface of heparinase III-treated Calu-3 monolayers. In contrast, only ∼30% of PAO1Δ*fliC*-GFP bound to HS-rich patches whereas ∼70% co-localized with HS-poor patches. In Calu-3 cells briefly treated with tunicamycin, only ∼40% of PAO1Δ*pilA*I-GFP co-localized to N-glycan-rich patches whereas a much larger fraction, almost 70%, of PAO1Δ*fliC*-GFP co-localized to N-glycan-rich areas (Figures 3C and D). In summary, PAO1Δ*pilA*, for which flagella serve as the major adhesin, preferentially co-localized with HS-rich areas. In contrast, PAO1Δ*fliC*, for which Tfp serve as the major adhesin, preferentially co-localized with N-glycan-rich areas at the AP surface of incompletely polarized epithelium.

We then tested whether Tfp or flagella were sufficient to mediate these specific interactions by quantifying the binding of Tfp- or flagella- coated green fluorescent beads to N-glycan or HSPG-rich areas at the AP surface. As shown in Figures 3E and F, 75% of flagella-coated beads co-localized with HS-rich areas at the AP surface of heparinase III-treated Calu-3 cells. In stark contrast, only 15% of Tfp-coated beads co-localized with HS-rich patches. The opposite results were observed for binding to N-glycans: ∼30% of flagella-coated beads compared to 80% of Tfp-coated beads co-localized with N-glycan-rich areas (Figures 3G and H). Importantly, the results paralleled what was observed with intact bacteria. Altogether, these experiments demonstrate that Tfp are sufficient to mediate *P. aeruginosa* binding to N-glycan chains and that flagella are sufficient to mediate *P. aeruginosa* binding to HS chains of HSPGs at the surface of airway epithelium.

### Bacterial internalization is mediated by Tfp-dependent binding to N-glycans at the AP surface or by flagella-dependent binding to HS at the BL surface of polarized epithelium

**Figure 4:**
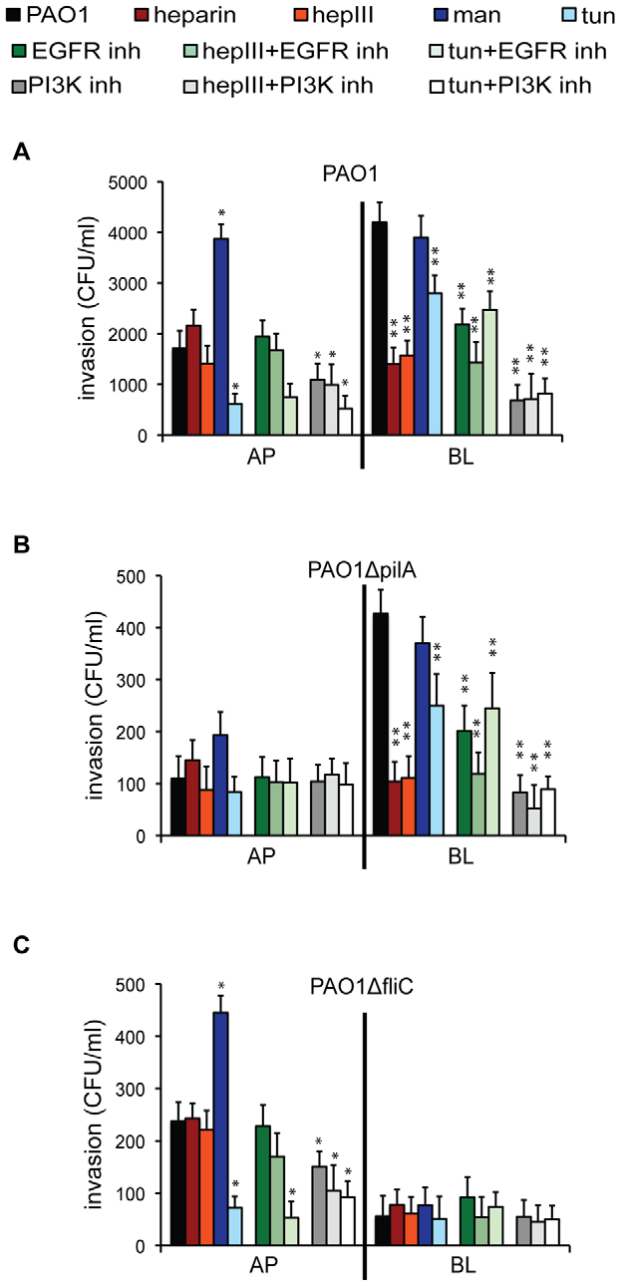
*P. aeruginosa* internalization at the AP surface of polarized epithelium is mediated by Tfp-dependent binding to N-glycans and subsequent PI3K activation and, at the BL surface, by flagella-dependent binding to HS and subsequent EGFR/PI3K activation. Calu-3 cells were grown as well polarized monolayers on Transwells for 9 days and treated with heparin, heparinase III (hepIII), mannose (Man), tunicamycin (tun), EGFR inhibitor (AG1478), PI3K inhibitor (LY29004), or in combination. (**A**) PAO1, (**B**) PAO1Δ*pilA* or (**C**) PAO1Δ*fliC* were added to the AP or BL chamber for 2 h and standard invasion assays were performed. Shown is the mean +/− SD for 4 independent experiments. ^*^P<0.05 compared to cells infected with PAO1 at the AP surface (black bar). ^**^P<0.05 compared to cells infected with PAO1 at the BL surface (black bar).

Following binding to the epithelium, *P. aeruginosa* is able to enter into non-phagocytic cells; this event is most readily detectable in ∼75% of clinical, environmental, and laboratory strains, including PAO1 or PAK, that do not secrete the potent phospholipase ExoU but that encode ExoS [31], [41]. In order to examine the role of flagella and Tfp interactions during entry at the AP or BL surface, polarized Calu-3 cells were pre-treated with various agents and standard bacterial invasion assays were performed. In general, the results of the invasion assays with PAO1, PAO1Δ*pilA*, and PAO1Δ*fliC* were directly proportional to adhesion assays (see Figure 1), i.e., more binding resulted in more invasion. Competitive inhibition with heparin or enzymatic cleavage of HS by heparinase III reduced PAO1 and PAO1Δ*pilA* invasion at the BL, but not AP surface, of polarized cells (Figures 4A and B). Up-regulation of N-glycosylation by long-term culture of Calu-3 cells in the presence of Man enhanced the internalization of PAO1 or PAO1Δ*fliC* at the AP surface of polarized cells, while inhibition of N-glycosylation with tunicamycin reduced bacterial entry at the AP surface of polarized Calu-3 cells (Figures 4A and C). Notably, inhibition of N-glycosylation had a small but statistically significant effect on PAO1 and PAO1Δ*pilA* entry at the BL surface, although it did not have any effect on bacterial binding. This finding suggests that flagella-dependent entry, but not binding, at the BL surface may be mediated by a yet unidentified N-glycosylated receptor(s). Together, these data confirm that *P. aeruginosa*-induced binding and subsequent entry into polarized epithelium are primarily mediated by Tfp-dependent binding to host N-glycans at the AP surface and by flagella-dependent binding to HS chains at the BL surface of polarized epithelium.

### Flagella-mediated bacterial entry at the BL surface and Tfp-mediated entry at the AP surface of polarized epithelium involve PI3K/Akt activation

We have previously shown that *P. aeruginosa* internalization at the AP surface of incompletely polarized MDCK cells is actin-dependent and requires activation of PI3K and its effector protein Akt [33]. We therefore determined whether N-glycans and/or HSPGs acted as host receptors upstream of this signaling pathway and whether bacterial Tfp and flagella were bacterial partners associated with the PI3K/Akt-dependent entry at the AP or BL surface of polarized lung airway epithelial cells. Pre-treatment with LY29004, an inhibitor of PI3K, did not affect PAO1 binding at either the AP or BL surface of fully polarized Calu-3 cells (Figure S4). However, it had a pronounced effect on bacterial invasion at the BL surface, reducing it ∼5 fold, and it had a smaller but statistically significant effect on invasion at the AP surface (Figure 4A.). PI3K-dependent entry at the BL surface required flagella binding to HS, as inhibition of PI3K, competitive inhibition with heparin, or heparinase-III treatment decreased PAO1Δ*pilA* entry similarly to PAO1, but had no effect on the already low levels of PAO1Δ*fliC* entry (Figures 4B and C). At the AP surface, inhibition of PI3K caused a small but statistically significant decrease in PAO1Δ*fliC* invasion. Simultaneous PI3K inhibition and tunicamycin treatment did not further reduce PAO1Δ*fliC* entry (Figure 4C), suggesting that PI3K-dependent invasion at the AP surface could require Tfp-mediated binding to N-glycans. Together, these results suggest that flagella-mediated binding at the BL surface leads to *P. aeruginosa* internalization through a PI3K-dependent pathway. At the AP surface, Tfp mediated entry through a PI3K-dependent entry can also occur, although consistent with our previously published results [9], BL entry is more efficient than AP entry.

**Figure 5:**
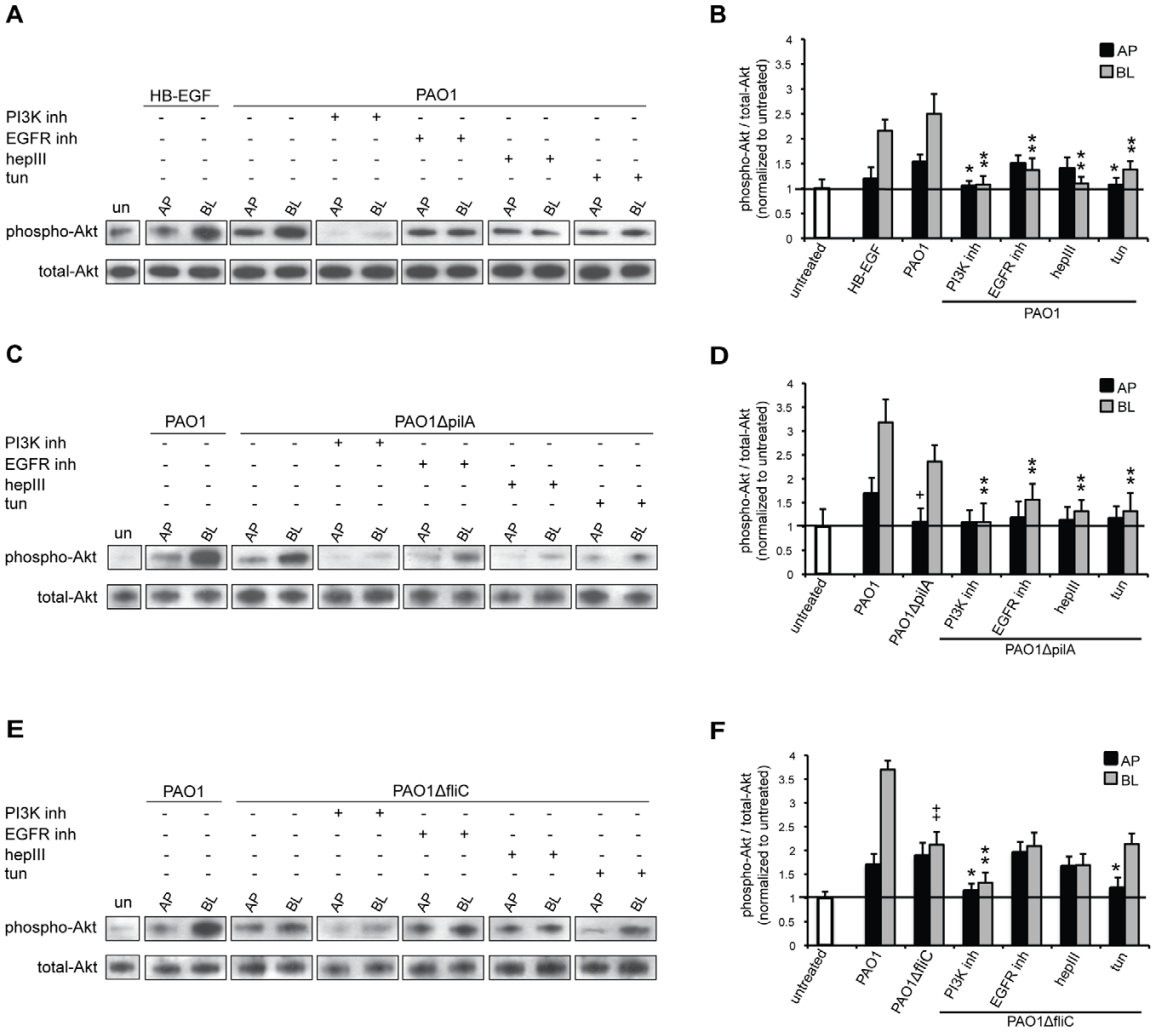
Akt is phosphorylated upon flagella-mediated *P. aeruginosa* entry at the BL surface and upon Tfp-mediated entry at the AP surface of polarized epithelium. Calu-3 cells were grown as well polarized monolayers on Transwells for 9 days and treated with heparinase III (hepIII), tunicamycin (tun), EGFR inhibitor (AG1478), or PI3K inhibitor (LY29004). As a control, cells were left untreated (un) or, as a positive control, cells were treated with HB-EGF. (**A**, **B**) PAO1, (**C**, **D**) PAO1Δ*pilA*, or (**E**, **F**) PAO1Δ*fliC* were added to the AP or BL chamber for 1 h. Lysates were immunoprecipitated with Akt antibody followed by immunoblotting with phospho- or total-Akt antibodies. Representative gels (A, C, E) and quantification by densitometry of three gels (B, D, F) are shown. The ratio of phospho-Akt to total-Akt for untreated cells was set to 1. Shown is the mean +/− SD for 3 independent experiments. ^+^P<0.05 compared to cells AP infected with PAO1. ^++^P<0.05 compared to cells BL infected with PAO1. ^*^P<0.05 compared to cells AP infected with PAO1 (B), PAO1Δ*pilA* (D), or PAO1Δ*fliC* (F). ^**^P<0.05 compared to cells BL infected with PAO1 (B), PAO1Δ*pilA* (D), or PAO1Δ*fliC* (F).

On the basis of our results, we would predict that flagella-mediated binding and entry at the BL surface or Tfp-mediated binding and entry at the AP surface should increase downstream Akt phosphorylation. To test this hypothesis, polarized Calu-3 cells were co-cultivated for 60 min with PAO1 or with adhesin mutants, and Akt was immunoprecipitated followed by immunoblotting with anti-phospho Akt^Ser473^ antibody. The ratio of phospho-Akt to total Akt was quantified and normalized to the ratio observed in untreated cells. In control experiments, Calu-3 cells were AP or BL exposed to heparin-binding EGF-like growth factor (HB-EGF) for 10 min since Akt phosphorylation is well established as a downstream consequence of EGFR activation. BL addition of HB-EGF increased Akt phosphorylation over 2-fold, but had little effect when added to the AP surface (Figures 5A and B), which is consistent with the known BL localization of EGFR in polarized epithelium and, thus, BL activation of Akt. Addition of PAO1 or PAO1Δ*pilA* at the BL surface resulted in activation of Akt, with a 2.5-fold increase in the fraction of phosphorylated Akt when compared to bacterial addition to the AP surface (Figures 5A–D). However, AP addition of PAO1Δ*pilA* or BL addition of PAO1Δ*fliC* failed to activate Akt (Figures 5C–F). This observation is consistent with a requirement for initial Tfp-mediated binding at the AP surface and flagella-mediated binding at the BL surface. Inhibition of PI3K prior to the addition of bacteria almost completely eliminated Akt phosphorylation at the AP and BL surface (Figures 5A–F), consistent with the known role of PI3K and Akt activation in *P. aeruginosa* entry [33].

We next determined whether Tfp or flagella-mediated activation of Akt involved HSPGs or N-glycans. Pre-treatment of Calu-3 cells with heparinase III reduced Akt phosphorylation to near basal levels upon BL, but not AP, addition of PAO1 or PAO1*ΔpilA* (Figures 5A–D). Inhibition of N-glycosylation with tunicamycin partially reduced Akt activation upon AP addition of PAO1 and PAO1*ΔfliC* (Figures 5A–B and E–F). Interestingly, inhibition of N-glycosylation reduced Akt phosphorylation upon BL infection with PAO1 or PAO1*ΔpilA*, which is consistent with our bacterial invasion results (see Figure 4) and suggests involvement of yet unidentified N-glycosylated receptor in flagella-HS-dependent invasion and activation of PI3K/Akt pathway. Together, these results strongly suggest that activation of PI3K/Akt pathway at the BL surface is primarily dependent on flagella-mediated bacterial binding to HS chains of HSPGs, while at the AP surface it is dependent on Tfp-mediated bacterial binding to N-glycan chains.

### Flagella-mediated *P. aeruginosa* entry at the BL surface involves EGFR activation

While many stimuli can activate the PI3K/Akt pathway, we were particularly interested in interrogating whether flagella- or Tfp-mediated binding to HSPGs or N-glycan chains and/or subsequent bacterial entry involved growth factor receptors (GFRs). Notably, GFRs require N-glycosylation for their activity and many of them are also modulated by HSPGs; our work clearly establishes the role of both N-glycans and HSPGs for *P. aeruginosa* binding and internalization.

In preliminary studies, we investigated the role of epidermal GFR (EGFR), platelet-derived GFR (PDGFR), and fibroblast GFR (FGFR). While pharmacologic inhibition of any of the GFRs did not affect bacterial binding, inhibition of EGFR and PDGFR, but not FGFR, reduced bacterial internalization at the BL surface of Calu-3 cells (Figure S4). We also confirmed the role of EGFR and PDGFR in *P. aeruginosa* entry using siRNA gene silencing in HeLa cells (Figure S4). Simultaneous removal of HS by heparinase III and pharmacologic inhibition of EGFR had the same effect as heparinase III treatment alone (Figure S4). These results suggest that EGFR may potentially mediate bacterial entry upon HS-dependent bacterial binding, and thus EGFR was a logical candidate to study further.

To elucidate the role of EGFR in flagella- and Tfp-mediated bacterial internalization, we first showed that inhibition of EGFR reduced invasion of PAO1 and PAO1Δ*pilA* at the BL surface of polarized Calu-3 cells, but did not further decrease the invasion of PAO1*ΔfliC* (Figure 4). Inhibition of EGFR did not have any effect on bacterial internalization at the AP surface since EGFR is predominantly expressed on the BL surface of polarized epithelium. Simultaneous inhibition of N-glycosylation and pharmacologic inhibition of EGFR reduced PAO1 and PAO1Δ*pilA* internalization at the BL surface similarly to what was observed with each treatment alone (Figures 4A and B), confirming that EGFR activity depends on its N-glycosylation. Inhibition of PAO1Δ*pilA* entry by heparin, heparinase III treatment, or PI3K inhibition reduced bacterial entry to similar degrees, while EGFR inhibition had somewhat intermediate effect (Figure 4B). Furthermore, combined heparinase III treatment and EGFR inhibition reduced PAO1 and PAO1Δ*pilA* internalization to a greater degree than inhibition of EGFR alone (Figures 4A and B). These results suggest that bacterial internalization occurs through multiple HSPG-dependent pathways, including one that involves a flagella-HS-EGFR complex leading to PI3K activation at the BL surface of polarized epithelium.

**Figure 6:**
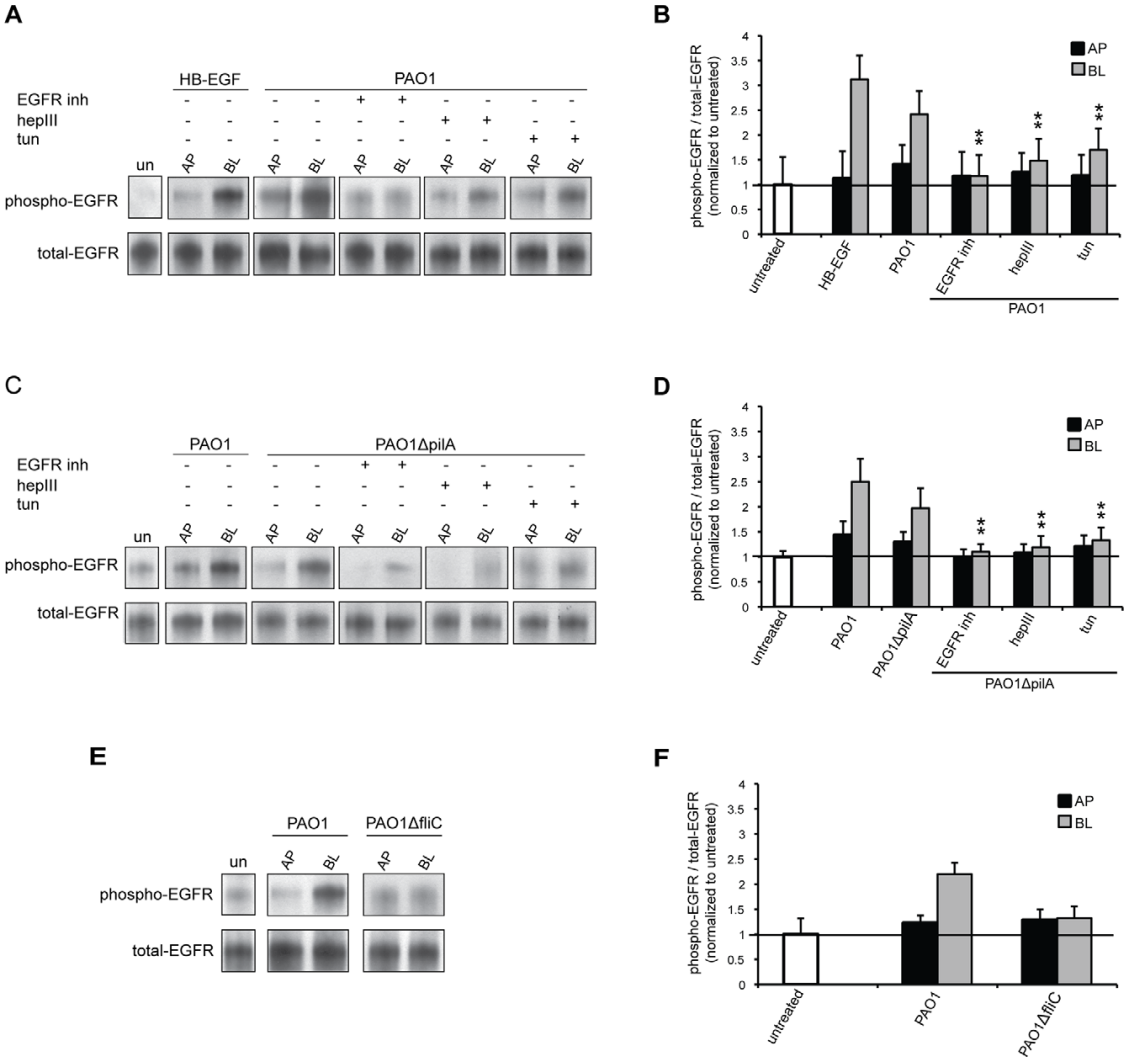
EGFR is phosphorylated upon flagella-mediated *P. aeruginosa* entry at the BL surface of polarized epithelium. Calu-3 cells were grown as well polarized monolayers on Transwells for 9 days and treated with heparinase III (hepIII), tunicamycin (tun), or EGFR inhibitor (AG1478). As a control, cells were left untreated (un) or, as a positive control, cells were treated with HB-EGF. (**A, B**) PAO1, (**C, D**) PAO1Δ*pilA*, or (**E, F**) PAO1Δ*fliC* were added to the AP or BL chamber for 1 h. Lysates were immunoprecipitated with EGFR antibody followed by immunoblotting with phospho- or total-EGFR antibodies. Representative gels (A, C, E) and quantification by densitometry of three gels (B, D, F) are shown. The ratio of phospho-EGFR to total-EGFR for untreated cells was set to 1. Shown is the mean +/− SD for 3 independent experiments. ^**^P<0.05 compared to cells BL infected with PAO1 (B) or PAO1Δ*pilA* (D).

To further investigate the role of flagella or Tfp during EGFR-dependent entry at the AP or BL surface of polarized epithelial cells, we tested whether bacterial binding induced EGFR phosphorylation. Total EGFR was immunoprecipitated from cell lysates 1 h after AP or BL infection with PAO1, PAO1Δ*pilA* or PAO1Δ*fliC*, followed by immunoblotting with a monoclonal anti-phospho EGFR^Ser1046/1047^ antibody. The ratio of phospho-EGFR to total EGFR was quantified and normalized to the ratio observed in untreated cells. In control experiments, BL exposure of Calu-3 cells to HB-EGF for 10 min increased EGFR phosphorylation 3-fold, but had little effect when applied to the AP surface (Figures 6A and B), consistent with the known BL localization of EGFR in polarized epithelium. Binding of PAO1 or PAO1Δ*pilA* to the BL surface of polarized epithelium resulted in a 2- to 2.5-fold increase in the fraction of phospho-EGFR (Figures 6A–D). In contrast, binding of PAO1Δ*fliC* failed to increase EGFR phosphorylation above background levels (Figures 6E and F), suggesting that flagella-mediated binding is required for EGFR activation. Activation of EGFR upon BL addition of PAO1 or PAO1*ΔpilA* was reduced by EGFR inhibition, removal of HS by heparinase III, or inhibition of N-glycosylation by tunicamycin, confirming the involvement of bacterial flagella, host HSPGs, and N-glycosylation in bacteria-mediated EGFR phosphorylation.

Finally, we tested whether flagella-mediated PI3K activation was EGFR dependent. Indeed, inhibition of EGFR decreased Akt phosphorylation in response to BL addition of PAO1 or PAO1Δ*pilA*, but not PAO1Δ*fliC* (Figures 5A–D). These results indicate that *P. aeruginosa*-mediated internalization at the BL surface occurs principally via flagella-mediated interactions through a pathway that utilizes HSPGs and that involves EGFR and PI3K/Akt activation. At the AP surface, Tfp mediates bacterial entry through a PI3K/Akt pathway that is independent of EGFR.

### Flagella are sufficient to induce EGFR phosphorylation

**Figure 7:**
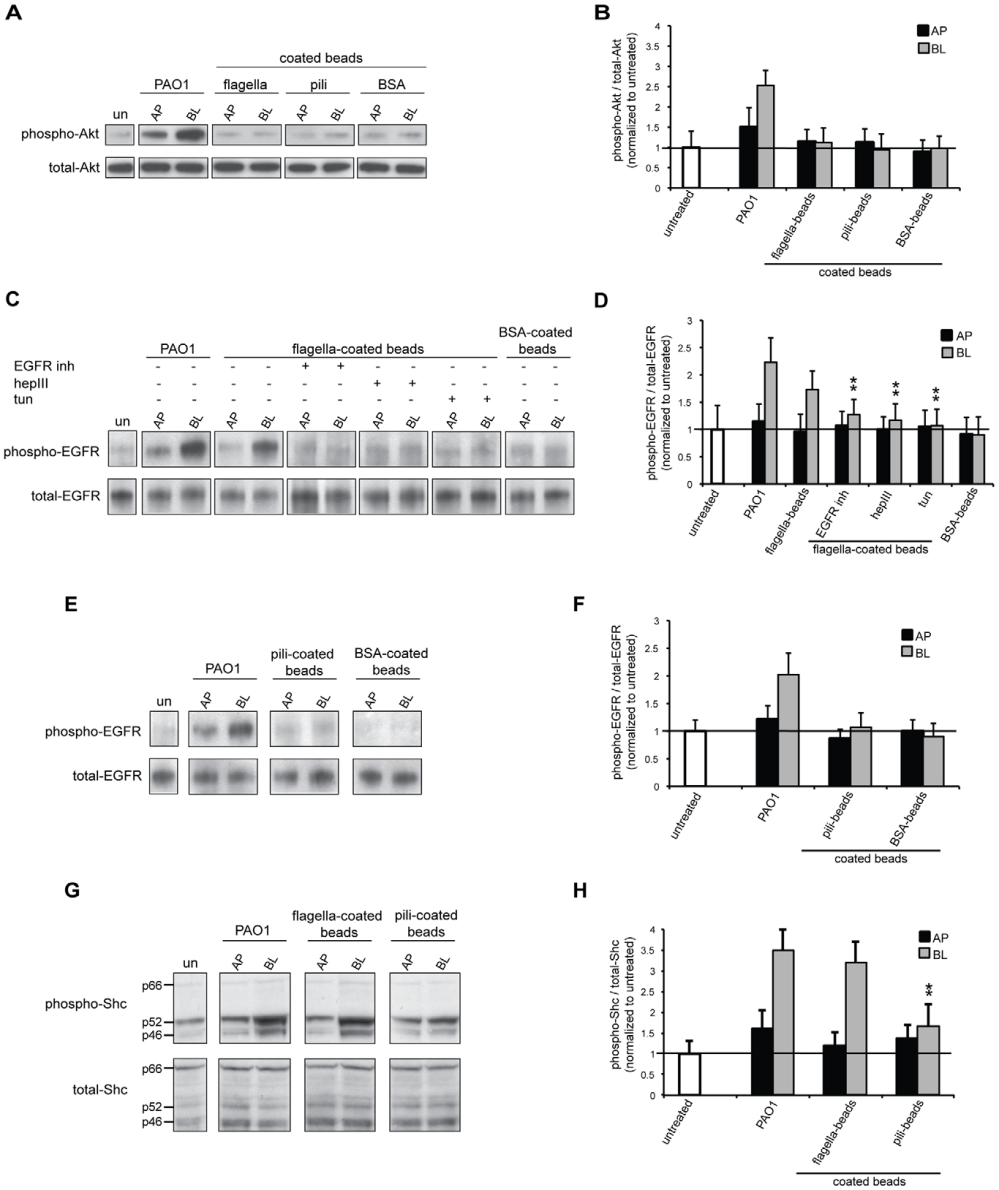
Flagella are sufficient to phosphorylate EGFR and Shc. Calu-3 cells were grown as well polarized monolayers on Transwells for 9 days and treated with heparinase III (hepIII), tunicamycin (tun), or EGFR inhibitor (AG1478). As a control, cell were left untreated (un); flagella-, Tfp-, or, as a negative control, BSA-coated beads were added to the AP or BL chamber for 1 h. Lysates were immunoprecipitated with Akt or EGFR antibody or directly immunoblotted with (**A, B**) phospho-Akt, (**C–F**) phospho-EGFR, or (**G–H**) phospho-Shc (three different isoforms p46, p52, and p66). Representative gels (A, C, E, G) and quantification by densitometry of three gels (B, D, F, H) are shown. The ratio of phospho-Akt to total-Akt, phospho-EGFR to total-EGFR, or phospho-Shc to total-Shc for untreated cells was set to 1. Shown is the mean +/− SD for 3 independent experiments. ^**^P<0.05 compared to cells BL infected with PAO1.

To elucidate whether Tfp and/or flagella alone were sufficient to induce phosphorylation of EGFR, beads coated with purified adhesins were added to polarized Calu-3 cells for 1 h and immunoprecipitation/immunoblotting assays were performed as previously for the whole bacteria. Remarkably, addition of flagella-coated beads to Calu-3 cells increased the fraction of phospho-EGFR (1.7-fold) (Figures 7 C and D), almost to levels seen upon BL addition of PAO1 (2.2-fold) (Figures 6 A and B). In contrast, no increase in the ratio of phospho-EGFR was observed upon addition of the flagella-coated beads to the AP surface or upon AP or BL addition of BSA-coated beads. Similar to what we observed with PAO1 or PAO1*ΔpilA*, flagella-mediated phosphorylation of EGFR was reduced after inhibition of EGFR, HS removal by heparinase III, or inhibition of N-glycosylation by tunicamycin (Figures 7C and D). Notably, AP or BL addition of Tfp-coated beads did not detectably increase phospho-EGFR above background levels (Figures 7E and F). We were unable to detect induction of Akt phosphorylation by either flagella- or Tfp-coated beads (Figures 7A and B). Altogether, these results demonstrate that flagella alone are sufficient to induce EGFR phosphorylation.

To confirm the specificity and significance of EGFR phosphorylation triggered by flagella-coated beads, we tested other targets of EGFR activation. As shown in Figures 7G and H, addition of flagella-coated beads to the BL surface of polarized epithelium resulted in phosphorylation of EGFR adaptor protein Shc (Src Homology-2 Domain Containing Transforming Protein) [42]. Shc exists in three isoforms (p46, p52, and p66) and we detected elevated levels of phosphorylated p46 and p52 isoforms upon infection with PAO1 or flagella-coated beads. In contrast, there was no increase in the ratio of phospho-Shc upon addition of the flagella-coated beads to the AP surface or upon AP or BL addition of pili-coated beads. Although, at this point, we are not able to show the activation of signaling events farther downstream of EGFR, such as Akt phosphorylation, these results confirm the significance of EGFR phosphorylation by flagella- but not pili-coated beads.

HB-EGF, but not EGF, needs to bind to HS chains of HSPGs to activate EGFR. Since our results demonstrate that flagella likewise activate EGFR in an HS-dependent manner, we first tested whether flagella-coated beads bind to EGFR or HB-EGF. While flagella-coated beads bound with great avidity to HS coated onto plastic wells, they did not bind measurably to the extracellular domain of EGFR, to HB-EGF, or to EGF, used as a negative control since it does not bind to HS to activate EGFR (data not shown). Second, we tested whether HB-EGF can inhibit binding of flagella-coated beads to HS chains. At a high concentration, exogenous HB-EGF, but not EGF, slightly inhibited binding of flagella-coated beads to HS coated onto plastic wells (Figure S5). These results suggest that, at high concentrations, HB-EGF can either compete with bacterial flagella for binding sites on HS chains or it sterically hinders flagella binding to HS.

## Discussion

Adhesion of pathogens to the host epithelium is an early and critical step in mucosal infections, and successful pathogens exploit specific niches to colonize, obtain nutrients, replicate, and disseminate. In previous studies utilizing well polarized epithelial cells, we have shown that the important nosocomial pathogen *P. aeruginosa* binds preferentially to different host molecules at the AP versus BL surface, specifically to N-glycans at the AP surface and to HSPGs at the BL surface [9]. We hypothesized that these complex *P. aeruginosa*-host interactions may be mediated by distinct bacterial adhesins. In the current studies, we identify the bacterial adhesins that are necessary and sufficient to mediate these different binding specificities. We demonstrate that Tfp are necessary to mediate maximal binding and entry at the AP surface through N-glycans, while flagella are required to mediate maximal binding and entry through HSPGs at the BL surface of polarized epithelium. While flagella have been shown previously to be required for the host response to BL infection with *P. aeruginosa* [43], [44], our studies using beads coated with purified Tfp or flagella are the first to demonstrate that Tfp or flagella are sufficient to mediate the differential binding to N-glycans or HSPGs, respectively.

Our studies reveal that flagella can also mediate, to a small extent, bacterial binding to N-glycans at the AP surface of polarized epithelium. However, we cannot determine at this point how significant these interactions are in *P. aeruginosa* infections due to the constrains of utilized assays; advanced more sensitive assays need to be employed to further investigate these interactions. Although we can detect N-glycan-dependent bacterial binding at the BL surface, neither up- nor down-regulation of N-glycosylation has any effect on the binding. Therefore, we postulate that binding of *P. aeruginosa* to N-glycan chains on the BL surface of polarized epithelium is not essential, rather the protein core of N-glycoprotein(s) play a role in the binding.

We investigated the consequences of these binding events on induction of host cell signal transduction pathways. We find that flagella-dependent bacterial binding to HS at the BL surface of polarized epithelium leads to activation of EGFR, as evidenced by increased phosphorylation of EGFR and of two of its associated downstream targets, the adaptor protein Shc and the serine/threonine kinase Akt. Strikingly, flagella alone are sufficient for EGFR and Shc phosphorylation upon binding of flagella-coated beads to the BL surface. Tfp-mediated bacterial binding at the AP surface also results in increased Akt phosphorylation, although the activation is less robust when compared to flagella-mediated bacterial binding at the BL surface of polarized epithelium.

Although we were able to detect elevated levels of phosphorylated EGFR and Shc upon binding of flagella-coated beads to the BL surface, we were unable to detect induction of Akt phosphorylation by either flagella- or Tfp-coated beads. Adhesin-coated beads may not be sufficient to trigger more downstream signaling events and additional bacterial factors may be required [31], [41]. Furthermore, adhesins coated onto beads may not be fully functional. Although we show that three-dimensional structure of isolated pili is most likely intact, Tfp extension/retraction is compromised and, thus, certain Tfp functions may be hindered when studied in the context of adhesin-coated beads. Finally, we cannot exclude that our detections assays are not sensitive enough to measure induction of downstream signaling events.

**Figure 8:**
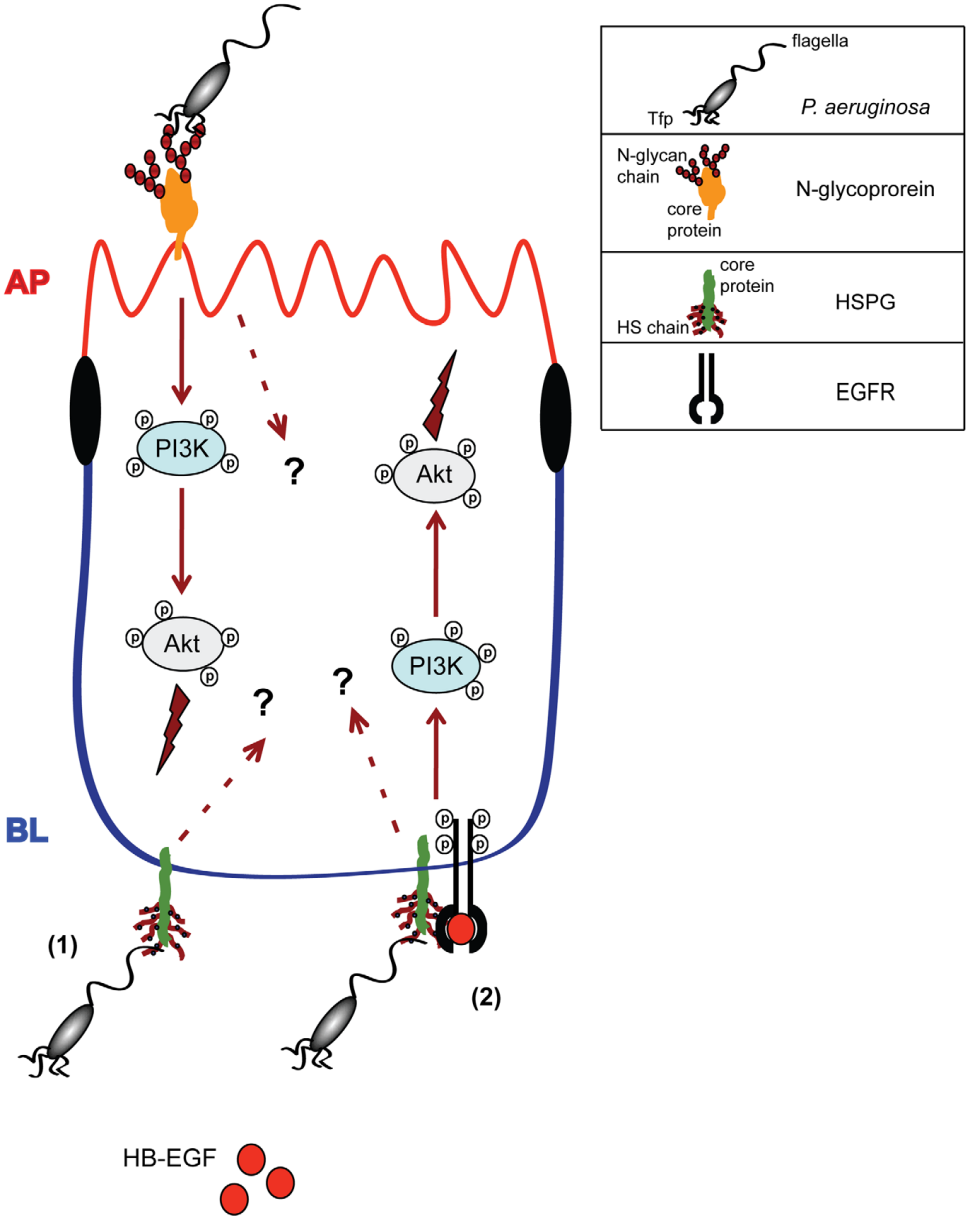
Model for Tfp- or flagella-mediated *P. aeruginosa* binding and induction of signaling pathways and events at the AP or BL surface of polarized epithelium. At the AP surface, *P. aeruginosa* binds to N-glycan chains through Tfp, which results in activation of PI3K and Akt. It remains to be investigated whether other signaling pathways can be activated upon Tfp binding to N-glycans. At the BL surface, (1) *P. aeruginosa* binds to HS chains of HSPGs through flagella and (2) the binding results in formation of the complex with EGFR and HB-EGF, which leads to activation of EGFR and subsequent activation of PI3K/Akt. Other signaling pathways can be possibly activated upon flagella binding to HS, dependently or independently of EGFR. Activation of PI3K/Akt at the AP surface, and EGFR and PI3K/Akt at the BL surface result in bacterial internalization and, most likely, in other pathogenic and host response events.

Based on our results, we propose a model (Figure 8), in which *P. aeruginosa* binds in a Tfp-dependent manner to N-glycan chains of one or more yet unidentified glycoproteins, which leads, to some extent, to the activation of PI3K/Akt pathway at the AP surface of polarized epithelium. It remains to be investigated whether other signaling pathways are also activated upon Tfp-mediated binding to N-glycans. At the BL surface, *P. aeruginosa* first binds in a flagella-dependent manner to HS chains of HSPGs. An attractive model is that a complex is formed between flagella, HPSGs, HB-EGF, and EGFR, which then leads to activation of EGFR and subsequent activation of the PI3K/Akt pathway. Flagella-mediated activation of EGFR most likely involves initial binding of flagella to HS chains of HSPGs since flagella-coated beads bind with great avidity to HS, but they do not bind measurably to HB-EGF or to the extracellular domain of EGFR. We attempted to discern whether flagella and HB-EGF compete for binding to HS; however, only a high concentration of an exogenous HB-EGF, far greater than concentrations required for EGFR phosphorylation *in vitro*, interferes with flagella binding to HS. Finally, we cannot exclude that other signal transduction pathways are activated upon flagella mediated binding to HSPGs, independent and/or dependent on EGFR phosphorylation.

One consequence of activation of EGFR and the PI3K/Akt pathway is *P. aeruginosa* internalization into host epithelium. Interestingly, inhibition of PI3K has a more pronounced effect on bacterial internalization at the AP surface of incompletely polarized epithelial cells (unpublished data and [33]) when compared to well polarized cells here studied. This phenomenon likely reflects differences in the composition of the AP versus BL surface during different stages of cell polarization. There may be increased levels of HSPGs on the AP surface of incompletely polarized cells [9]; in addition, there may be differences in how *P. aeruginosa*-mediated pathogenic events are affected by changes in the levels of host receptors that occur during the polarization process (unpublished data and [9]).

Bacterial binding and activation of EGFR and the PI3K/Akt pathway most likely lead to other pathogenic events as well. We have recently shown that inhibition of bacterial binding to N-glycans at the AP surface and to HS at the BL surface reduces *P. aeruginosa*-mediated host damage [9]. Similarly, pharmacologic inhibition of EGFR reduces bacterial cytotoxicity at the BL surface and inhibition of PI3K reduces bacterial toxicity at both the AP and BL surface of polarized epithelium (unpublished data). However, it has been shown that *P. aeruginosa* infection of incompletely polarized corneal epithelial cells that leads to EGFR activation through shedding of the HB-EGF ectodomain, followed by activation of ERK1/2 and PI3K pathways, results in inhibition of apoptosis in the early stage of bacterial infection [45]. Thus, further studies are needed to elucidate the role of HSPGs- and HB-EGF-dependent EGFR and PI3K/Akt pathways in *P. aeruginosa*-mediated cell death.

Activation of EGFR and other growth factors is an emerging theme in bacterial pathogenesis. Multiple pathogens have been shown to activate EGFR, including *Neisseria gonorrhea*, *Neisseria meningitides*, *Helicobacter pylori*, *Pasteurella multocida*, and *Haemophilus influenzae* [46]–[52]. Of particular relevance are studies with *N. gonorrhea*, which, similar to *P. aeruginosa*, binds to the AP surface of polarized epithelial cells as microcolonies that initiate changes in the host cell actin cytoskeleton and allow the microcolonies to enter into epithelial cells. Upon binding, EGFR is phosphorylated, its activation is required for *N. gonorrhea* internalization, and phospho-EGFR is found in close apposition to a fraction of surface bound microcolonies [49], [52]. We were unable to determine by IF microscopy whether phospho-EGFR co-localized with bound flagella-expressing *P. aeruginosa* or with flagella-coated beads, because of high background from staining with the anti-phospho-EGFR antibody. Nonetheless, the similarities between these two organisms are intriguing.

Our finding of Tfp- and flagella-dependent binding to N-glycans at the AP surface and HSPGs at the BL surface of polarized epithelium, respectively, and subsequent EGFR/PI3K/Akt signaling events introduce another level of complexity to diverse mechanisms of *P. aeruginosa* adhesion and establishment of an infection. Several N-glycosylated receptors have been identified, including the Cystic Fibrosis Transmembrane Regulator (CFTR), fibronectin, or integrins [53], [54]. However, since fibronectin and integrins are preferentially expressed at the BL surface, their N-glycan chains are unlikely to be AP receptors for bacterial Tfp. Although CFTR is expressed on the AP surface of polarized epithelium [53], it is also unlikely that N-glycan chains of CFTR mediate Tfp-dependent bacterial binding under the conditions of our experiments, as we observed similar levels of bacterial adhesion to the AP surface of epithelial cells that express either very low or high amounts of CFTR ([9] and unpublished data). Previous studies have also suggested that glycosphingolipids, i.e., asialoGM1, may serve as AP receptors for Tfp [22], [23]; however, glycosylation of sphingolipids differs from N-glycosylation and, thus, Tfp binding to N-glycans characterized in this paper most likely represents a distinct mechanism by which *P. aeruginosa* is able to infect the host.

Flagella, likely through its interaction with TLR5, have been shown to activate the innate immune response preferentially at the basolateral surface of polarized airway epithelial cells [43], [44]. Utilizing informative flagella mutants or purified flagellin, it has been possible to uncouple TLR5-mediated NFκB-dependent inflammatory responses from EGFR-dependent epithelial cell proliferation, wound repair, and antimicrobial peptide production [55]. TLR5 activates EGFR through signaling events that are not dependent on HSPGs [56] and, thus, TLR5 is very unlikely to be involved in flagella- and HSPGs-dependent cascade leading to EGFR phosphorylation. TLR5 is predicted to be N-glycosylated [57] and since we show that modulation of N-glycosylation does not affect flagella-mediated binding to the BL surface, N-glycan chains of TLR5 most likely do not mediate binding of bacterial flagellin. Since expression of most TLRs can be variable [58] and we can detect low flagella-dependent *P. aeruginosa* binding to N-glycans on the AP surface of polarized epithelium, we cannot exclude that in certain pathogenic conditions *P. aeruginosa* may bind in a flagella-dependent manner to N-glycan chains of TLR5 on the AP surface.

Other bacterial factors have been implicated in mediating *P. aeruginosa* binding to the host. *P. aeruginosa* LPS binds to TLR4, predominantly expressed on the BL surface of polarized epithelium, and it has been reported to stimulate human lung epithelial wound repair through a TLR4- and EGFR-dependent pathway that involves release of the EGFR ligand, TGF-α, by the matrix metalloprotease TACE [59]. Flagellar components have been shown to bind to Lewis^X^ derivatives found on secreted mucins [19] and *P. aeruginosa* can additionally stimulate mucin secretion in an EGFR-dependent manner, as shown in rat tracheal cells [60]. Furthermore, two different *P. aeruginosa* lectins, PA-IL (LecA) and PA-IIL (LecB), have been implicated in bacterial binding to sugar moieties present on mucins or cell surface receptors [36]. Work from our lab and others have implicated more receptors and signaling pathways in *P. aeruginosa* entry or host responses to bacterial infection, including PDGFR, Abl/Crk [31], and Src family kinases [61], e.g. Lyn [62], and it will be of interest to determine if any of these molecules are differently activated upon Tfp- or flagella-mediated binding at the AP or BL surface of polarized epithelium.

In summary, *P. aeruginosa* can utilize numerous adhesins or virulence factors and exploit numerous host receptors to adhere to the host epithelium. Studies focused on providing key insights into multifactorial and complex *P. aeruginosa* binding are crucial for comprehensive understanding of this event and identification of potential therapeutic targets. Our findings introduce bacterial and host players and link them with previously described signaling events to build novel network of interactions and events that lead to establishment of *P. aeruginosa* acute or chronic infections. Tfp and flagella as well as corresponding glycosylated host receptors are potentially valuable targets for designing therapies that interfere with the initial steps in *P. aeruginosa* infection and colonization. Such therapies could also target a number of other carbohydrate-based interactions of *P. aeruginosa* with the host, including bacterial binding to mucus and biofilm formation [32], [36]. Therefore, these anti-adhesive therapies are a very attractive strategy for development of novel treatments for a wide range of both acute and chronic *P. aeruginosa* infections.

## Materials and Methods

### Bacterial strains

*P. aeruginosa* strain O1 (PAO1) was obtained from the ATCC (ATCC 15692) and isogenic mutants PAO1Δ*pilA* and PAO1Δ*fliC* were previously constructed in the laboratory [63]. PA103 [64] was a kind gift of Dr. Dara Frank and PA103 Mutant 9 was previously constructed in the laboratory [38]. All strains were routinely grown shaking overnight in Luria-Bertani broth (LB broth) at 37°C. GFP-expressing strains were created by electroporation of the pnpT2-GFP-pUCP20 plasmid as described previously [9].

### Bacterial adhesion and invasion assays

Following overnight growth in LB broth at 37° with shaking, bacteria were added to well polarized cells at an MOI of 20. For AP infections, the bacteria in serum-free MEM were added to the AP chamber of cells grown on Transwells. For BL infections, the Transwell insert was placed directly onto 50 µl of serum-free MEM containing bacteria. After 2 h of infection at 37°C, adhesion and invasion assays were performed as described previously [9]. Bacteria were enumerated by plating serial dilutions of cell lysates to LB plates and counting colony-forming units (cfu). All assays were carried out on triplicate wells, and results are reported as the average of three to five experiments.

### Isolation of flagella and Tfp

To isolate surface flagella or Tfp, PAO1Δ*pilA*, PAO1Δ*fliC*, PA103, or PA103 Mutant 9 were grown shaking overnight in 2 ml LB at 37°C and 100 µl of the culture was plated onto 1.5% LB agar. Following growth at 37°C for 16 h, the bacteria were scraped from the agar surface and resuspended in 5 ml PBS. A volume of cells equivalent to an optical density at 600 nm (OD_600_) of 20.0 was resuspended in 1 ml PBS. Cells were vortexed vigorously at room temperature for 30 min to remove surface flagella or Tfp by shearing. The suspension was centrifuged at 20,000× *g* for 10 min at 4°C, the supernatant was collected and centrifuged a second time to remove all cellular debris. The resulting supernatant was dialyzed against PBS (pH 7.4) overnight at 4°C and centrifuged at 20,000× *g* for 20 min at 4°C to remove insoluble proteins. Afterward, the supernatant was incubated overnight at 4°C in 100 mM MgCl_2_ to precipitate flagella or Tfp. The precipitate was collected by centrifugation at 4°C (15,000× *g* for 20 min), and the pellet was resuspended and dialyzed against PBS (pH 7.4) overnight at 4°C. Afterward, the suspension was centrifuged at 20,000× *g* for 20 min at 4°C to remove insoluble proteins, and flagella or Tfp were precipitated again in 100 mM MgCl_2_ at 4°C. Dialysis, centrifugation, and precipitation steps were repeated again to obtain flagella or Tfp of a high purity as assessed by SDS-PAGE and staining in 0.25% Coomassie (Bio-Rad) for 4 h. Destaining was done in 10% ethanol, 7.5% acetic acid for 6 h or until bands appeared and the background was clear.

### Preparation of adhesin- or sugar-coated fluorescent beads

For adsorption of bacterial adhesins to beads, 0.5 ml of 2.5% suspension of Green Fluoresbrite Latex fluorescent beads (2 µm ∅; Polysciences Inc.) were mixed with 200 µg of purified Tfp or flagella in 0.1 M Borate Buffer overnight at room temperature according to the manufacturer's protocol. To determine the coating efficiency, coated beads were eluted in SDS sample buffer and analyzed by Western blotting. Following SDS-PAGE and transfer, the membranes were probed with a 1∶100,000 dilution of primary α-FliC (for flagella) or α-PilA (for Tfp) [63] antibody overnight at 4°C, followed by probing with a 1∶25,000 dilution of horseradish peroxidase-conjugated secondary antibody (Jackson ImmunoResearch Laboratories). Gels were quantified by densitometry using ChemiDoc XRS and coating efficiencies were calculated. On average, 15–20% (30 µg) flagella and ∼30% (60 µg) Tfp were bound to the beads. For adsorption of glycosylated molecules to beads, 0.5 ml of Red Fluoresbrite Latex fluorescent beads (2 µm ∅; Polysciences, Inc.) were mixed with 400 µg of HS, N-glycan-3, Man, or Gal according to the manufacturer's protocol. Bead coating efficiency (30–40%) was determined by eluting coated beads in SDS sample buffer, dotting an aliquot on a Zeta-Probe membrane (Bio-Rad), staining the membrane with 1% Toluidine Blue, and comparing the staining to standards.

### Cell culture

Calu-3 cells were obtained from the ATCC and maintained in MEM supplemented with 10% fetal bovine serum (FBS; Invitrogen) and L-glutamate at 37°C with 5% CO_2_. Cells were grown as 2D monolayers on 12-mm Transwell filters (3-µm pore size; Corning Inc.) as previously described [9]. For experiments, Calu-3 cells were seeded at 1.5×10^6^ cells/well and cultured for 3 days (“incompletely polarized monolayers”) or at 1×10^6^ cells/well on Transwells and cultured for 9 days (“well polarized monolayers”). Under each condition, cells formed polarized confluent monolayers as determined by polarized distribution of some AP and BL membrane proteins and the presence of functional tight junctions that were impermeable to small molecules such as FITC-inulin (data not shown and [9]). However, in incompletely polarized monolayers, distribution of HSPGs on the BL surface was not fully polarized [9].

### Enzymatic and inhibitory treatments

To remove glycosaminoglycans, Calu-3 cells were treated with 200 mU of heparinase III or chondroitinase ABC (Sigma-Aldrich) in Hank's Buffered Salt Solution (HBSS) containing 0.1% BSA at 37°C for 2 h (resulting in 60–65% reduction in glycosaminoglycan expression), or, for partial removal of glycosaminoglycans, with 50 mU of heparinase III for 30 min (resulting in ∼25% reduction in expression). To assess the efficiency of treatments, HS chains were visualized by IF staining with HS antibody (10E4; Seikagaku), CS chains were visualized with FITC-WFA (CS-specific lectin from *Wisteria floribunda*; Sigma-Aldrich), and staining densities were quantified using ImageJ and compared to the staining densities of untreated cells (data not shown and [9]). For competition blocking experiments with glycosaminoglycans, cells were pre-incubated with 5 µg/ml of heparin or CS (Sigma-Aldrich) at 37°C for 1 h in serum-free MEM. For up-regulation of N-glycosylation, cells were grown in the presence of 1 mM Man or Gal (Sigma-Aldrich) in MEM with 10% FBS for 1 week (resulting in 1.4-to-1.7 fold increase in N-glycosylation). To inhibit N-glycosylation, cells were pre-treated with 1 µg/ml of tunicamycin (Sigma-Aldrich) for 16 h (∼50% reduction) or for 8 h (brief de-glycosylation resulting in ∼20% reduction) in MEM with 10% FBS. To assess cell surface N-glycosylation, cells were stained with the Man-specific lectin FITC-concanavalin A (Sigma-Aldrich), staining densities were quantified using ImageJ and compared to the staining densities of untreated cells (data not shown and [9]). To inhibit EGFR, PDGFR, or FGFR, cells were pre-incubated with 10 µM AG1478, AG1296, or PD173074 (Calbiochem) in MEM with 10% FBS for 1 h. To inhibit PI3K, cells were pre-incubated with 50 µM LY294002 (Sigma-Aldrich) in MEM with 10% FBS for 1 h. Inhibition efficiencies were quantified by Western blotting using phospho-specific antibodies.

### Protein depletion by siRNA

EGFR (sc-29301), PDGFR-α (sc-29443), PDGFR-β (sc-29442), and control (sc-37007) siRNAs were purchased from Santa Cruz Biotechnology. HeLa cells (ATCC CCL-2), grown in MEM supplemented with 10% FBS, were transfected with siRNAs according to the manufacturer's instructions. After 42 h, standard adhesion and invasion assays were performed. In parallel, lysates were immunoblotted with appropriate antibodies to evaluate the efficiency of protein depletion.

### *In vitro* binding assays

HSPGs, glycosaminoglycans, N-glycan-1, -2, and -3, and sugars (Sigma Aldrich; 0.1–10 µg in 0.2 ml ddH_2_O) were added to 96-well polystyrene plate (Corning) and incubated overnight at 37°C until evaporated. Wells were washed with ddH_2_O and blocked in 0.1% BSA for 0.5 h at room temperature. Bound molecules were stained with 1% Toluidine blue (Sigma-Aldrich) and absorbance was measured at 630 nm. The absorbance of known concentration of molecules was used as the standard curve and the concentration of bound molecules (µg/well) was calculated. 100 µl of flagella- or Tfp-coated beads in ddH_2_O were added to coated wells and incubated for 2 h on a rotary shaker. Non-adherent beads were removed by washing with ddH_2_O. For some experiments, 5–20 ng/ml HB-EGF or EGFR were added to heparin-coated wells before addition of flagella-coated beads. Bound beads was quantified using a SpectraMax 340PC plate reader using SOFTmaxPro software (Molecular Devices) at λex = 480 nm and λem = 530 nm. Beads bound to non-coated wells were used as a control and subtracted out as background. The results are reported as the average of six experiments, each with at least 6 replicates.

For the bead-bead aggregation assay, red fluorescent beads, 100 µl of green fluorescent beads coated with flagella or Tfp were allowed to aggregate with 100 µl of red fluorescent beads coated with various glycosylated molecules on a rotary shaker at 50 rpm for 2 h in ddH_2_O. For competition blocking experiments, 5 µg/ml of heparin or N-glycan-3 (Sigma-Aldrich) were added to wells. Images of aggregates were acquired with a confocal microscope (LSM 510; Carl Zeiss MicroImaging, Inc.) equipped with a 20× objective. Image processing was performed using Adobe Photoshop CS4 version 11.0.2. Quantification of the fraction of green, red, and mixed (yellow) aggregates from 3 separate experiments and 10 events per each sample was performed using UTHSCSA Image Tool version 2.00 Alpha.

### Immunoprecipitation and immunoblotting

Well polarized Calu-3 cells grown on Transwells for 9 days were washed and placed in serum-free MEM for ∼17 h. Bacteria at the MOI of 200 or 50 µl adhesin-coated beads were added to the AP or BL chamber for 1 h. As a control, 10 ng/ml HB-EGF was added to the AP or BL-chamber for 10 min. The infected and HB-EGF-treated monolayers were washed with cold PBS containing 1 mM sodium orthovanadate (Sigma-Aldrich). Cells were lysed in modified radioimmunoprecipitation (RIPA) buffer (50 mM Tris, pH 7.4, 150 mM NaCl, 2 mM EDTA, 2 mM EGTA, 1% Triton X-100, 0.5% deoxycholate, 0.1% SDS, 1 mM sodium orthovanadate, 50 mM NaF, 0.1 mM okadaic acid (Sigma-Aldrich), 1 mM phenylmethylsulfonyl fluoride (Sigma-Aldrich), and proteinase inhibitor tablets (Complete; Roche Diagnostics)) for 20 min, and cells were removed from the Transwell filters by gentle scraping. The cell lysates were centrifuged at 16,000× *g* for 20 min. Immunoprecipitation with Akt or EGFR antibody (Cell Signaling Technology) using Magnetic Dynabeads Protein G beads (Invitrogen) were performed according to the manufacturer's protocol. For detection of Shc, whole cell lysates were used without immuprecipitation. Cell lysates or eluted immune complexes were separated on Novex-NuPAGE 10% Bis-Tris SDS-PAGE gels (Invitrogen) and electroblotted to iBlot Nitrocellulose Membranes using the iBlot Device (Invitrogen) according to the manufacturer's protocol. Membranes were blocked in PBS containing 0.05% Tween 20 and 5% non-fat milk (PBST) and probed with a 1∶1000 dilution of an antibody that recognizes Akt phosphorylated on serine 473, EGFR phosphorylated on serine 1046/1047, or Shc phosphorylated on tyrosine 239/240 (Cell Signaling Technology) in PBST, overnight at 4°C. Membranes were then incubated with a 1∶3000 dilution of horseradish peroxidase-conjugated secondary antibody (Jackson ImmunoResearch Laboratories) and detected by enhanced chemiluminescence using the Amersham ECL Western blotting detection kit (GE Healthcare). For loading control, membranes were stripped and re-probed with an antibody that recognizes all forms of Akt, EGFR, or Shc (Cell Signaling Technology). Gels were quantified by densitometry using ChemiDoc XRS.

### Immunofluorescence microscopy and image analysis

Calu-3 cells grown on Transwells as incompletely polarized monolayers were infected with PAO1Δ*pilA*-pGFP, PAO1Δ*fliC*-GFP (MOI 50), or with 50 µl flagella- or Tfp-coated green fluorescent beads for 2 h at room temperature. Afterwards, cells were washed and fixed in PBS containing 1% paraformaldehyde at 37°C for 0.5 h. After washing, cells were incubated with primary antibodies overnight at 4^ø^C and, afterwards, with fluorescent secondary antibodies for 2 h at room temperature. HS chains were stained with 1∶500 anti-heparan sulfate antibody (10E4; Seikagaku) followed by 1∶2,000 AlexaFluor647-conjugated secondary antibody (Invitrogen). Actin filaments were stained with 1∶2,000 AlexaFluor594-phalloidin (Invitrogen) and Man residues were detected by staining with 1∶1,000 FITC-conjugated lectin concanavalin A (Sigma-Aldrich). Filters were excised and mounted on microscope slides (Fisher Scientific) in mounting medium (Vector Laboratories, Inc.). Samples from 3 separate experiments and 20 events per each sample were examined with a confocal microscope (LSM 510; Carl Zeiss MicroImaging, Inc.). Images and 3D reconstructions were acquired by and processed in Meta 510 software. Image J analysis was performed on TIFF files. Bacterial or bead binding to Calu-3 cells and co-localization with surface markers was quantified using the Image J plugin Voxel counter on 3D reconstructions of TIFF images acquired with Meta 510 software. Voxel Counter (ImageJ plugin) was used to quantify the volume of bound 3D bacterial or bead aggregates and a minimum volume was set as a threshold to enable automated cell counting using the 3D Object Counter (ImageJ plugin). Any aggregate above the threshold was counted as one. The surface area of membrane regions either enriched or depleted of HS or N-glycans (as determined by staining with an anti-HS antibody or with FITC-ConA, respectively) was measured in pixels by ImageJ, and the number of bacterial or bead aggregates bound was normalized per pixel of each specific surface area. The percentage (compared to total) of bacterial or bead aggregates bound to each specific region was determined.

### Statistical analysis

Data are expressed as means ± SD (standard deviation). Statistical significance was estimated by ANOVA test using InStat version 3.0b. Differences were considered to be significant at P<0.05.

## Supporting Information

Figure S1 **Purity of isolated flagella and Tfp preparations.** (**A**) Isolated flagella or Tfp from PAO1Δ*pilA* or PAO1Δ*fliC*, respectively, were coated onto fluorescent beads. The total amount used for coating, the supernatant fraction, and the bead-bound portion were separated by SDS-PAGE and immunoblotted with a polyclonal antibody to FliC (flagella) or to PilA (Tfp). (**B**) SDS-PAGE gel stained by Coomassie Blue.
(TIF)

Figure S2 **Flagella-coated beads bind directly to HS and Tfp-coated beads bind directly to N-glycans** ***in vitro*** **.** Isolated flagella or Tfp from PAKΔ*pilA* or PAKΔ*fliC*, respectively, were coated onto green fluorescent beads. 96-well plastic plates were coated overnight with increasing concentrations of the indicated molecules. (**A**) Flagella- or (**B**) Tfp-coated beads were added to 96-well plastic plates coated with increasing concentrations of various molecules for 1 h. The fluorescence of the bound fraction was quantified in a plate reader and the percent of binding above control (binding of coated beads to non-coated wells) is indicated. Shown is the mean +/− SD for 6 independent experiments. HSPGs: heparan sulfate proteoglycans, HS: heparan sulfate; HA: hyaluronic acid; CS-4: 4-0-sulfated chondroitin sulfate; CS-6: 6-0-sulfated chondroitin sulfate; ; N-glycan-1: simple N-glycan chain; N-glycan-2: hybrid N-glycan chain; N-glycan-3: complex N-glycan chain; Man: mannose; GlcNAc: N-acetylglucosamine; Fuc: fucose; Gal: galactose.
(TIF)

Figure S3 **The C-terminus of Tfp is required for binding of coated beads to N-glycans.** Tfp were isolated from PA103 or PA103 Mutant 9 separated on (**A**) 12% SDS-PAGE gel stained by Coomassie Blue. Tfp isolated from (**B**) PA103 or (**C**) PA103 Mutant 9 were coated onto green fluorescent beads and added to 96-well plastic plates coated with increasing concentrations of various molecules for 1 h. The fluorescence of the bound fraction was quantified in a plate reader and the percent of binding above control (binding of coated beads to non-coated wells) is indicated. Shown is the mean +/− SD for 3 independent experiments. HSPGs: heparan sulfate proteoglycans, HS: heparan sulfate; HA: hyaluronic acid; CS-4: 4-0-sulfated chondroitin sulfate; CS-6: 6-0-sulfated chondroitin sulfate; N-glycan-1: simple N-glycan chain; N-glycan-2: hybrid N-glycan chain; N-glycan-3: complex N-glycan chain; Man: mannose; GlcNAc: N-acetylglucosamine; Fuc: fucose; Gal: galactose.
(TIF)

Figure S4 ***P. aeruginosa*** **internalization, but not adhesion, is dependent on EGFR, PDGFR, and PI3K.** Calu-3 cells were grown as well polarized monolayers on Transwells for 9 days and treated with heparinase III (hepIII), EGFR inhibitor (AG1478), PDGFR inhibitor (AG1296), FGFR inhibitor (PD173074), PI3K inhibitor (LY29004), or in combination. PAO1 was added to the AP or BL chamber for 2 h and (**A**) standard adhesion or (**B**) invasion assays were performed. (**C**) PAO1 invasion in HeLa cells after siRNA depletion of EGFR, PDGFR-α, and PDGFR-β. Shown is the mean +/− SD for 3 independent experiments. ^*^P<0.05 compared to BL infected cells (black bar) in panels A and B or to control in panel C.
(TIF)

Figure S5 **High concentrations of HB-EGF compete with binding of flagella-coated beads to HS** ***in vitro*** **.** Flagella isolated from PAO1Δ*pilA* were coated onto green fluorescent beads and 96-well plastic plates were coated with 5 µg/well HS. Increasing concentrations of HB-EGF or EGFR were added to HS-coated wells, followed by addition of flagella-coated beads for 1 h. The fluorescence of the bound fraction above control (flagella-coated beads bound to non-coated wells) was quantified in a plate reader and normalized to flagella-coated beads bound to HS-coated wells (set to 1). Shown is the mean +/− SD for 3 independent experiments.
(TIF)
