# Supplementary figures and images for: Pseudomonas aeruginosa Pili and Flagella Mediate Distinct Binding and Signaling Events at the Apical and Basolateral Surface of Airway Epithelium
Source: PLoS Pathog. 2012 Apr 5;8(4):e1002616. doi: 10.1371/journal.ppat.1002616 (PMC3320588; doi:10.1371/journal.ppat.1002616)

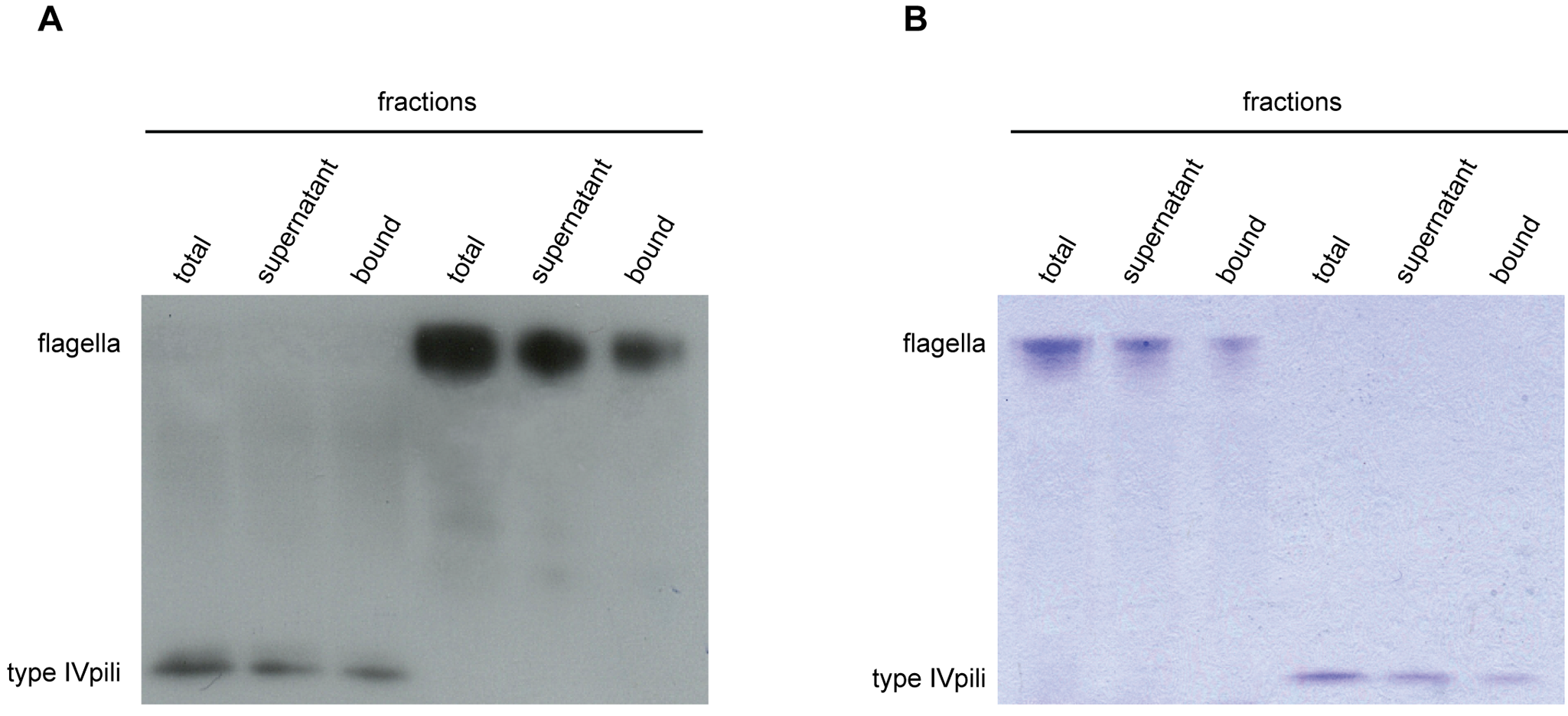

Supplement: Figure S1 — Purity of isolated flagella and Tfp preparations. (A) Isolated flagella or Tfp from PAO1ΔpilA or PAO1ΔfliC, respectively, were coated onto fluorescent beads. The total amount used for coating, the supernatant fraction, and the bead-bound portion were separated by SDS-PAGE and immunoblotted with a polyclonal antibody to FliC (flagella) or to PilA (Tfp). (B) SDS-PAGE gel stained by Coomassie Blue. (TIF) [file ppat.1002616.s001.tif]

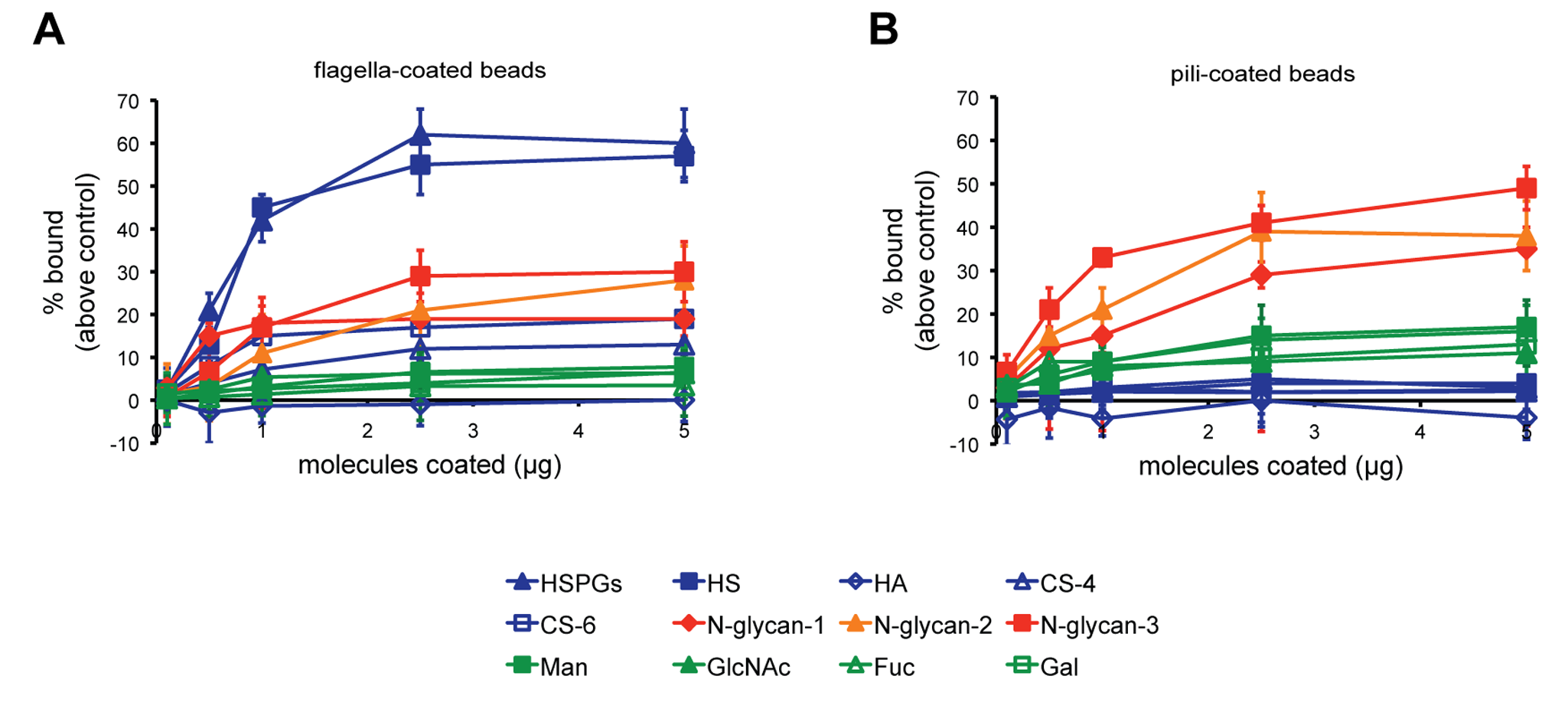

Supplement: Figure S2 — Flagella-coated beads bind directly to HS and Tfp-coated beads bind directly to N-glycans in vitro . Isolated flagella or Tfp from PAKΔpilA or PAKΔfliC, respectively, were coated onto green fluorescent beads. 96-well plastic plates were coated overnight with increasing concentrations of the indicated molecules. (A) Flagella- or (B) Tfp-coated beads were added to 96-well plastic plates coated with increasing concentrations of various molecules for 1 h. The fluorescence of the bound fraction was quantified in a plate reader and the percent of binding above control (binding of coated beads to non-coated wells) is indicated. Shown is the mean +/− SD for 6 independent experiments. HSPGs: heparan sulfate proteoglycans, HS: heparan sulfate; HA: hyaluronic acid; CS-4: 4-0-sulfated chondroitin sulfate; CS-6: 6-0-sulfated chondroitin sulfate; ; N-glycan-1: simple N-glycan chain; N-glycan-2: hybrid N-glycan chain; N-glycan-3: complex N-glycan chain; Man: mannose; GlcNAc: N-acetylglucosamine; Fuc: fucose; Gal: galactose. (TIF) [file ppat.1002616.s002.tif]

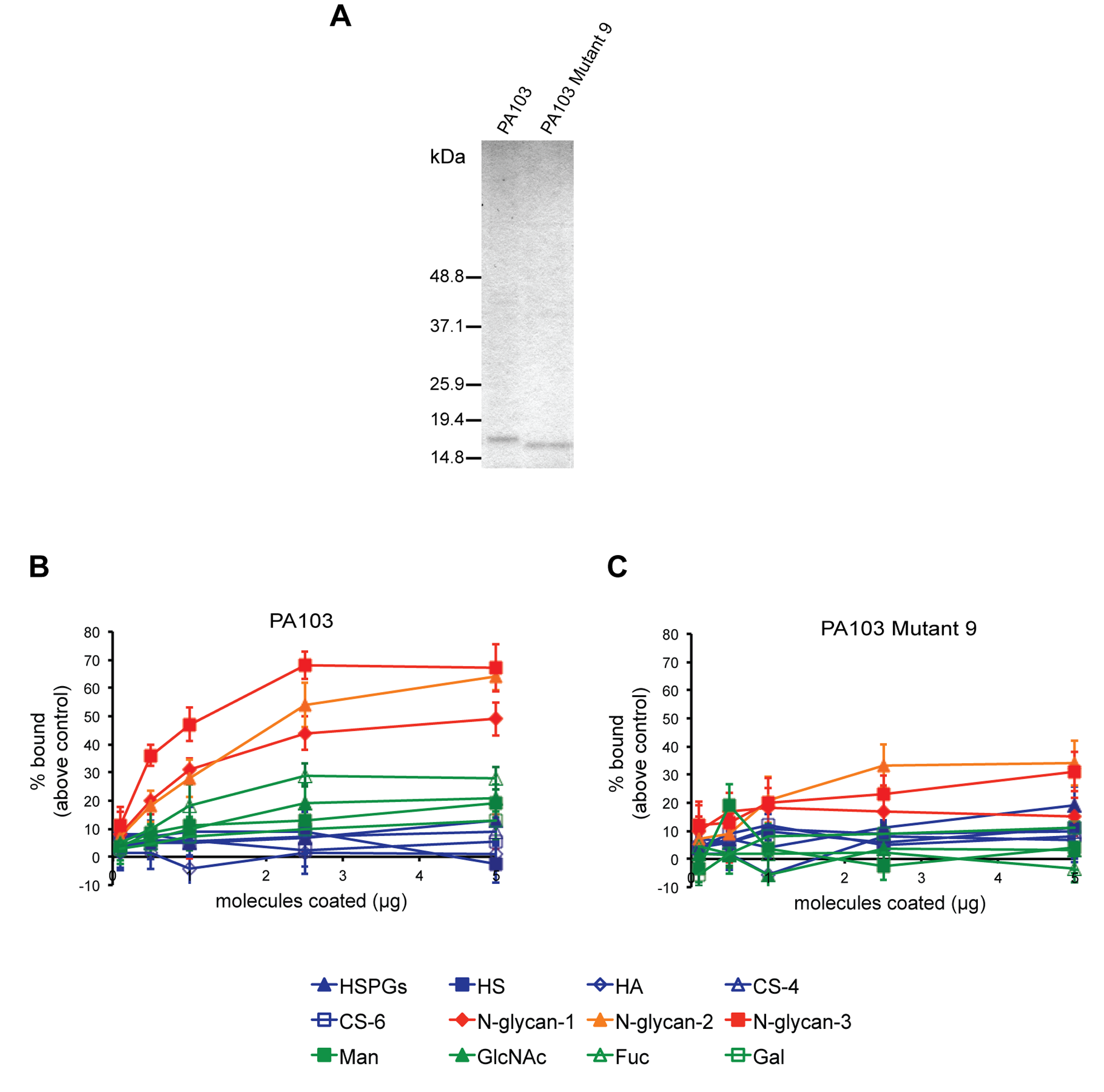

Supplement: Figure S3 — The C-terminus of Tfp is required for binding of coated beads to N-glycans. Tfp were isolated from PA103 or PA103 Mutant 9 separated on (A) 12% SDS-PAGE gel stained by Coomassie Blue. Tfp isolated from (B) PA103 or (C) PA103 Mutant 9 were coated onto green fluorescent beads and added to 96-well plastic plates coated with increasing concentrations of various molecules for 1 h. The fluorescence of the bound fraction was quantified in a plate reader and the percent of binding above control (binding of coated beads to non-coated wells) is indicated. Shown is the mean +/− SD for 3 independent experiments. HSPGs: heparan sulfate proteoglycans, HS: heparan sulfate; HA: hyaluronic acid; CS-4: 4-0-sulfated chondroitin sulfate; CS-6: 6-0-sulfated chondroitin sulfate; N-glycan-1: simple N-glycan chain; N-glycan-2: hybrid N-glycan chain; N-glycan-3: complex N-glycan chain; Man: mannose; GlcNAc: N-acetylglucosamine; Fuc: fucose; Gal: galactose. (TIF) [file ppat.1002616.s003.tif]

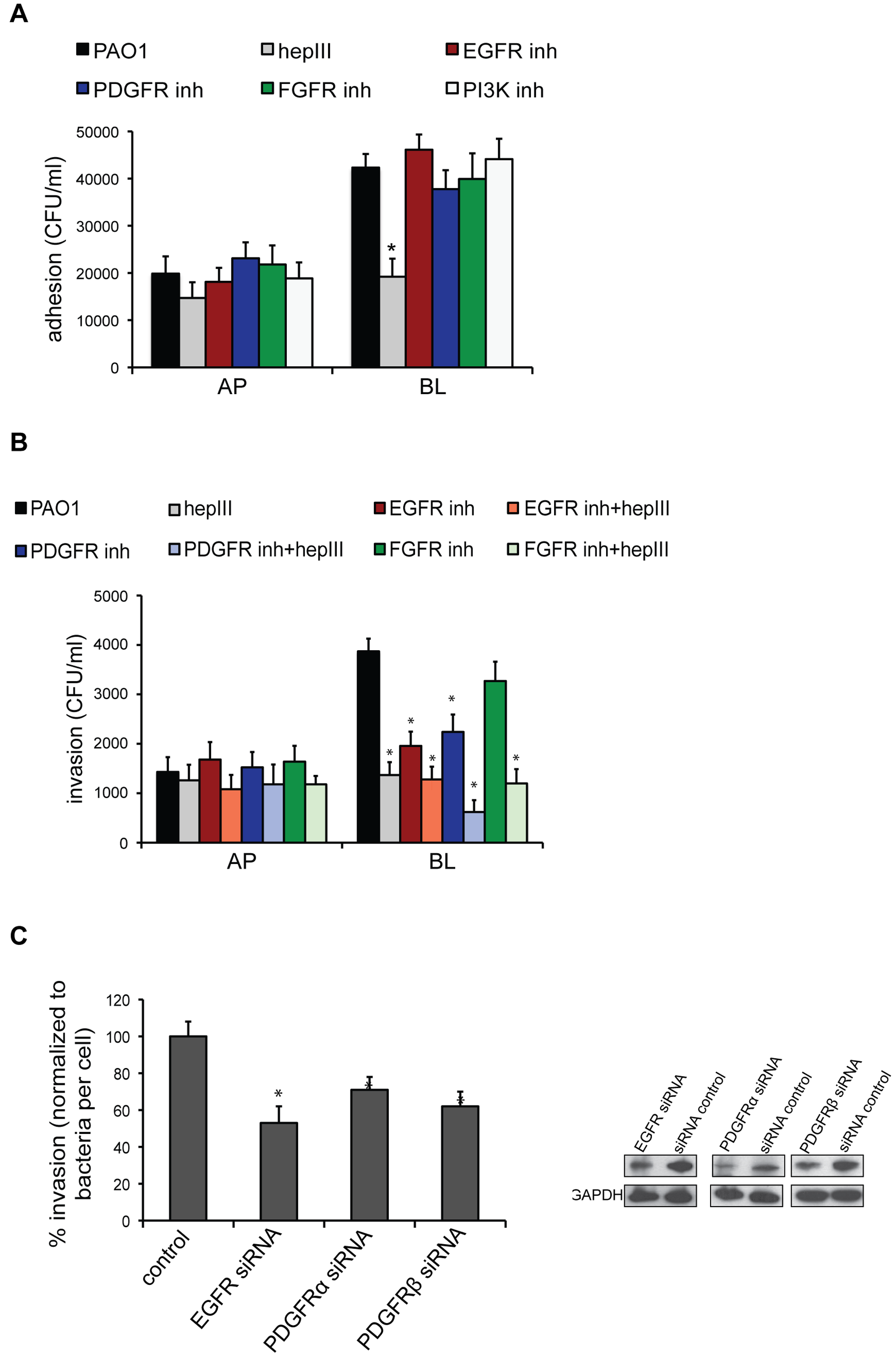

Supplement: Figure S4 — P. aeruginosa internalization, but not adhesion, is dependent on EGFR, PDGFR, and PI3K. Calu-3 cells were grown as well polarized monolayers on Transwells for 9 days and treated with heparinase III (hepIII), EGFR inhibitor (AG1478), PDGFR inhibitor (AG1296), FGFR inhibitor (PD173074), PI3K inhibitor (LY29004), or in combination. PAO1 was added to the AP or BL chamber for 2 h and (A) standard adhesion or (B) invasion assays were performed. (C) PAO1 invasion in HeLa cells after siRNA depletion of EGFR, PDGFR-α, and PDGFR-β. Shown is the mean +/− SD for 3 independent experiments. *P<0.05 compared to BL infected cells (black bar) in panels A and B or to control in panel C. (TIF) [file ppat.1002616.s004.tif]

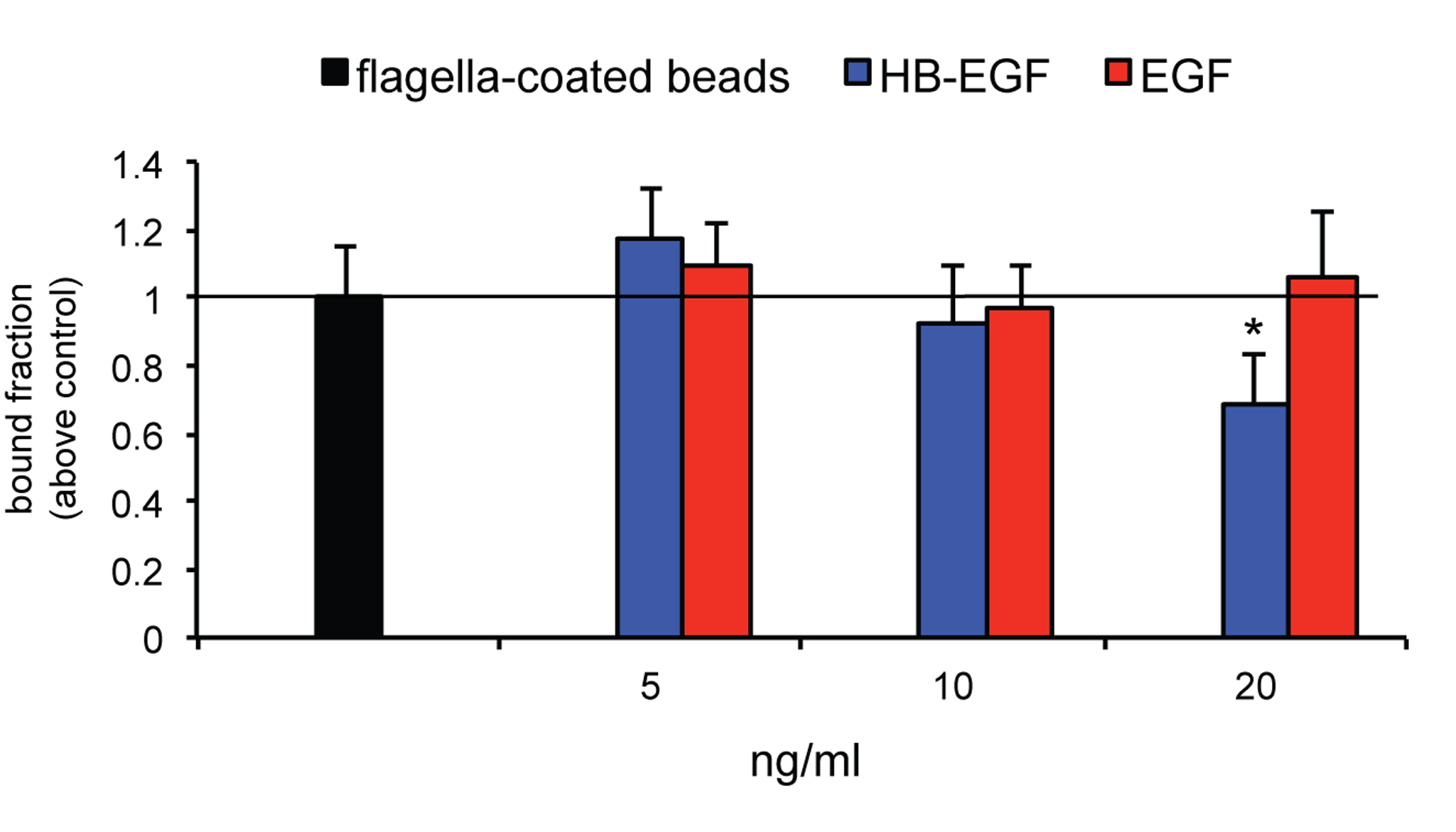

Supplement: Figure S5 — High concentrations of HB-EGF compete with binding of flagella-coated beads to HS in vitro . Flagella isolated from PAO1ΔpilA were coated onto green fluorescent beads and 96-well plastic plates were coated with 5 µg/well HS. Increasing concentrations of HB-EGF or EGFR were added to HS-coated wells, followed by addition of flagella-coated beads for 1 h. The fluorescence of the bound fraction above control (flagella-coated beads bound to non-coated wells) was quantified in a plate reader and normalized to flagella-coated beads bound to HS-coated wells (set to 1). Shown is the mean +/− SD for 3 independent experiments. (TIF) [file ppat.1002616.s005.tif]
